# Environmental Modulation of Mini-Clonal Gardens for Cutting Production and Propagation of Hard- and Easy-to-Root *Eucalyptus* spp

**DOI:** 10.3390/plants11233281

**Published:** 2022-11-29

**Authors:** Johnatan Vilasboa, Cibele T. Da Costa, Arthur G. Fett-Neto

**Affiliations:** 1 Plant Physiology Laboratory, Federal University of Rio Grande do Sul, Porto Alegre, RS 91501-970, Brazil; 2 Center for Biotechnology, Federal University of Rio Grande do Sul, Porto Alegre, RS 91501-970, Brazil; 3 Department of Botany, Federal University of Rio Grande do Sul, Porto Alegre, RS 91501-970, Brazil

**Keywords:** adventitious rooting, propagation by cutting, shoot branching, *Eucalyptus*, mini-tunnel, environmental modulation, seasonality, gene expression, mineral nutrition

## Abstract

Clonal *Eucalyptus* propagation is essential for various industry sectors. It requires cuttings to successfully develop adventitious roots (ARs). Environmental conditions are influential on AR development and may be altered to modulate the productivity of hard-to-root clones. The current knowledge gap in research on the physiological patterns underlying commercial-scale propagation results hinders the design of novel strategies. This study aimed to identify patterns of variation in AR-relevant parameters in contrasting seasons and species with distinct rooting performances. *E. dunnii* and *E.* ×*urograndis* (hard- (hardR) and easy-to-root (easyR), respectively) mini-stumps were subjected to light modulation treatments and to mini-tunnel use (MT) for a year. The treatment impact on the branching and rooting rates was recorded. The carbohydrate content, AR-related gene expression, and mineral nutrition profiles of cuttings from the control (Ctrl) and treated mini-stumps were analyzed. Light treatments were often detrimental to overall productivity, while MTs had a positive effect during summer, when it altered the cutting leaf nutrient profiles. Species and seasonality played large roles in all the assessed parameters. *E.* ×*urograndis* was particularly susceptible to seasonality, and its overall superior performance correlated with changes in its gene expression profile from excision to AR formation. These patterns indicate fundamental differences between easyR and hardR clones that contribute to the design of data-driven management strategies aiming to enhance propagation protocols.

## 1. Introduction

From pulp and paper to timber and energy, several industry sectors depend on high-quality woody biomass provided by planted forests. As the most abundantly planted tree genus worldwide (over 20 million ha), *Eucalyptus* cultivation is a thriving economic activity. In Brazil alone, it accounts for 78% of a national forestry sector worth over USD 21 billion in 2020 [1]. Young forests such as these also have relevant carbon sequestration potential [2]. Planted *Eucalyptus* forests greatly benefit from clonal propagation by cuttings, mainly using the mini-cutting technique [3]. It consists of the establishment of clonal mini-gardens populated by donor plants, called mini-stumps, from which (mini-)cuttings are periodically (typically once a week) excised. The cuttings are then generally transferred to a shaded greenhouse, where they may develop roots in individual plastic tubes. The rooted propagules are then acclimated and shipped to the final cultivation sites [4]. Propagation by cuttings ensures the uniform development and maintenance of the elite quality of the planted genotypes [5]. A pivotal step in cutting propagation is the de novo formation of roots from cut stems, i.e., adventitious rooting. However, the competence required to undergo this developmental process varies among *Eucalyptus* species, and sometimes even among clones from the same species. For the forestry sector, this becomes an issue when a *Eucalyptus* clone/accession of interest (e.g., disease-resistant, frost-tolerant) has low rooting competence. Therefore, rooting recalcitrance can severely impair or even render unfeasible the cutting propagation of highly relevant genotypes.

Adventitious roots (ARs), defined as roots formed from non-root organs, evolved as part of a developmental plasticity toolkit that allowed plants to better respond to environmental changes such as flooding, nutrient deficiency, and mechanical damage [6]. Such a complex feature of plant development requires an intricate and dynamic regulation that can integrate internal signals and environmental cues [7]. AR formation is also remarkably sensitive to each specific triggering stimulus and, as mentioned above, genetic background [7]. Hormonal control, a key aspect of adventitious rooting, is known to differ between intact and cut stems [6,8,9].

In the case of cuttings, upon excision, a series of events take place both locally and systemically, which will be determinants of successful AR development. An almost immediate hormonal wound response is followed by an auxin maximum established at the cutting base due to an interruption in the basipetal flow of its polar transport. This leads to the activation of invertases and sink establishment, cell differentiation, and division [10,11]. With proper carbohydrate provision from the source tissues, the stem origin tissue (usually the cambium) can begin root primordia formation by undergoing tightly regulated division and differentiation steps [12]. Once fully formed, the AR primordia must emerge from the stem, aided by ethylene-stimulated cell wall loosening and programmed cell death [13]. The path from cutting excision to AR formation is not always direct. Callus formation at the cutting base has been reported for several species. In some of these, callus formation always precedes AR development [14]. With indirect organogenesis, however, comes the possibility of producing friable ARs, i.e., ARs without proper vascular connection with the cutting stem. In *Eucalyptus* cutting propagation, the presence of callus-derived ARs, or simply frequent callus formation, may be indicative of rooting recalcitrance.

Proper and timely auxin action is essential for AR development. This is achieved by a balance between the biosynthesis, transport, perception, signaling, and inactivation modules. The analysis of *Arabidopsis thaliana* (Arabidopsis) mutants has shed light on several auxin-related genes whose function is linked to adventitious rooting [12,15,16,17]. In *Eucalyptus*, in vitro studies have investigated AR-related gene expression profiles associated with light modulation [18], age-associated loss of rooting competence [19], and differences between species with different rooting rates [20]. However, the lack of such studies applied to commercial settings limit their use in drawing novel clonal garden management strategies.

While internal signals are major players in AR development, the environmental control of mini-stump (cutting donor plant) and mini-cutting growth conditions has been studied to overcome roadblocks in *Eucalyptus* propagation [5]. Of note are the effects of light quality and irradiance, air humidity and temperature, mineral nutrition [21], and water availability [5,22]. The enrichment of the far-red region of the light spectrum increased the rooting competence of *Chrysanthemum* cuttings [23] and recalcitrant *E. globulus* [24] micro-cuttings grown in vitro. Changes in fertilization regimes have also been shown to increase rooting rates in recalcitrant *Eucalyptus* species both in vitro [25] and at the commercial level [26]. More recently, the use of plastic mini-tunnels (MTs) has been shown to enhance mini-stump branching rates and cutting AR development in *Eucalyptus* spp. [27,28,29].

Given this context and the growing interest in *Eucalyptus* clonal forests in subtropical regions [1,30], the present study aimed to identify the general variation patterns of AR-relevant parameters associated with different seasons, clones, and environmental conditions that might indicate optimal propagation results. Morphophysiological aspects of cuttings obtained from control and treated hard-to-root (hardR) *Eucalyptus dunnii* and easy-to-root (easyR) *E.* ×*urograndis* mini-stumps were monitored in a commercial setting. Seasonality was found to play a major role in the different performances of each species. The *E.* ×*urograndis* cuttings underwent a unique temporal shift in their gene expression patterns that may be associated with the species’ higher productivity. A leaf nutrient profile analysis revealed differences between the species that may also account for their distinct productivity.

## 2. Results

Three *Eucalyptus* clones (genetic accessions) of varying rooting competences were subjected to environmental modulation treatments, namely far-red enrichment (FR), shading (SH), and mini-tunnel coverage (MT). During four seasons, the impacts of each treatment on the overall propagation, when compared to the control (Ctrl) plants, were monitored by collecting mini-stump branching and cutting rooting data. Samples were also collected during AR development for biochemical and molecular analyses.

### 2.1. Branching, Rooting, and Overall Productivity

**Figure 1:**
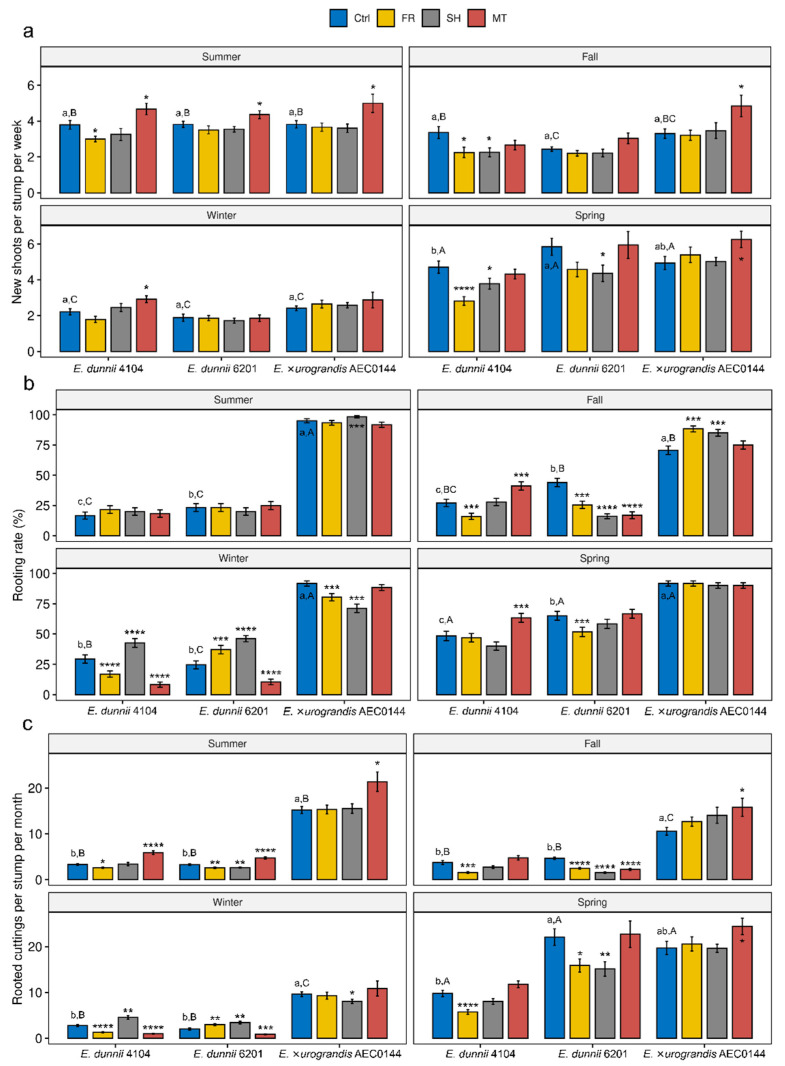
Impact of mini-stump environmental modulation on the propagation of *Eucalyptus dunnii* 4104, 6201 (hardR clones), and *E.* ×*urograndis* AEC0144 (easyR) across seasons. (**a**) Number of weekly new shoots per mini-stump (21 > *n* > 15), (**b**) rooting rates 30 days after excision (%, 60 > *n* > 26), and (**c**) monthly rooted cuttings per stump (21 < *n* < 15) during summer, fall, winter, and spring under control (Ctrl), far-red-enriched (FR), shade nets (SH), and mini-tunnel (MT) conditions. Bars represent mean ± SEM. Asterisks indicate significant differences in the treatments in comparison to the Ctrl (* *p* ≤ 0.05, ** *p* ≤ 0.01, *** *p* ≤ 0.001, **** *p* ≤ 0.0001, Dunnett’s test). Different lowercase and uppercase letters indicate statistical significance when comparing the Ctrl results across clones in the same season and Ctrl results across seasons in the same clone, respectively (factorial ANOVA, estimated marginal means test, *p* ≤ 0.05).

All the treatments were able to modulate branching rates of mini-stumps in a season- and clone-dependent manner (Figure 1a). However, the light-related treatments (FR and SH) were generally neutral or detrimental to branching, particularly for the hardR *E. dunnii* clones. The MT treatment had either a neutral effect or significantly positive effect on the weekly number of new branches. In the summer, MT-treated mini-stumps from all the clones had higher branching rates than the Ctrl ones. Branching seemed to be strongly impacted by seasonality, and all the clones showed reduced rates under control conditions during winter when compared to summer and spring. Furthermore, during winter, branching rates were generally unaffected by the three treatments. During most of the year, the branching rates of all three clones were similar to those of Ctrl plants within each season. In spring, however, significant variation was found between the hardR 4104 and 6201 Ctrl plants (Figure 1a). In the same season, the branching rates were highest for all three clones.

In contrast to the branching data, the colder seasons showed a larger range of rooting rate modulation impacts, although these were often associated with a negative effect (Figure 1b). During summer and spring, the rooting rates were largely unaffected. FR- and SH-derived effects varied considerably, as observed for easyR AEC0144 in the colder seasons.

Throughout the year, easyR AEC0144 cuttings had higher rooting success than hardR *E. dunnii* clones. Apart from the fall season, easyR AEC0144 had uniformly high rooting rates. Among hardR clones, 6201 had generally higher rooting competence than 4104, except in the winter. These clones reached their top rooting performance in spring.

In a commercial setting such as the one in which this study was conducted, the combined effects of the branching and rooting rates, or overall productivity, are what determines the propagation viability of a given *Eucalyptus* clone. To address how changes in branching and rooting affect overall productivity, a combined metric is presented in Figure 1c. It represents the mean number of excised branches that are expected to root within 30 days of cutting excision according to the seasonal mean rooting rate.

In the summer, the MT-treated mini-stumps from all the clones showed an increased overall productivity. The same effect was observed for easyR AEC0144 in the fall and spring. Conversely, the overall productivity of the MT-treated plants was impaired in the winter for both hardR *E. dunnii* clones. Light modulation generally resulted in a diminished overall productivity, mainly due to its negative effect on branching. Even so, exceptionally, for hardR *E. dunnii* in the winter, some positive effect could be observed. For most of the year, the superior rooting performance of easyR AEC0144 led to a higher overall productivity. In the spring, however, the combined overall increased branching and rooting rates of hardR 6201 resulted in productivity results comparable to those of easyR AEC0144. For all clones, general productivity peaked in spring and was lowest in winter. The performance of easyR AEC0144 in this aspect varied the most across seasons, decreasing during the colder seasons and recovering in spring.

The cutting chlorophyll content did not vary significantly with environmental modulation (Figure S1). In the hardR clones, seasonal variation was detected, with the colder seasons generally registering lower levels.

### 2.2. Developmental Outcomes of Cuttings and AR System Morphology

**Figure 2:**
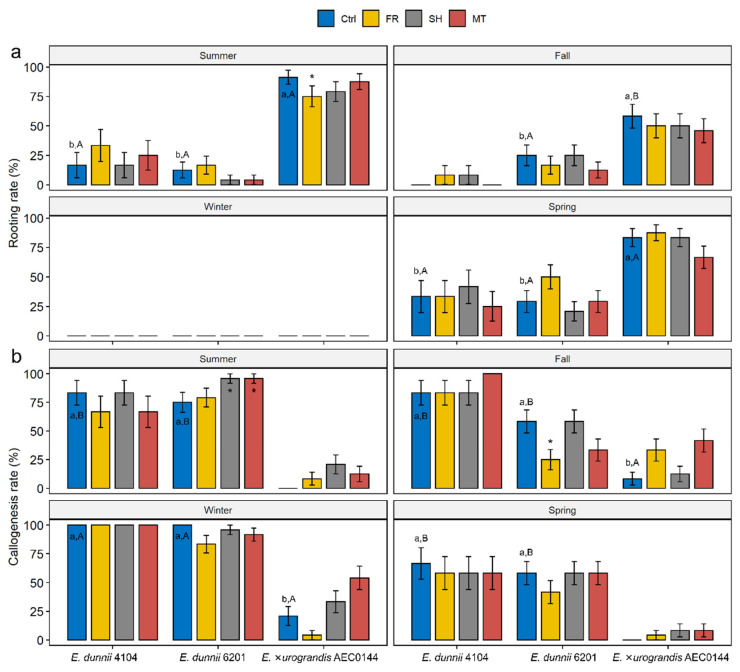
Developmental outcomes of *Eucalyptus dunnii* 4104 and 6201 (hardR clones) (at 21 days post-excision) and *E.* ×*urograndis* AEC0144 (easyR) cuttings (at 14 days post-excision). (**a**) Relative frequency (%) of rooted cuttings and (**b**) cuttings with calli, obtained from mini-stumps under control (Ctrl), far-red-enriched (FR), shade nets (SH), and mini-tunnel (MT) conditions. Bars represent mean ± SEM. Asterisks indicate significant differences in the treatments in comparison to the Ctrl (* *p* ≤ 0.05, Dunnett’s test). Different lowercase and uppercase letters indicate significant divergence when comparing Ctrl results across clones in the same season and Ctrl results across seasons in the same clone, respectively (ANOVA, Tukey’s test, *p* ≤ 0.05).

In addition to the rooting evaluation at 30 dpe, AR development was also monitored weekly from excision to 21 dpe. At 7 dpe, ARs rarely formed in the hardR clones, often only reaching measurable lengths at 21 dpe. In contrast, the easyR AEC0144 cuttings frequently developed ARs by 14 dpe. The developmental rate discrepancy can be observed in Figure S2. Therefore, these two timepoints were chosen for the interspecific comparisons. Figure 2 illustrates the relative frequency of the two more common outcomes observed at these timepoints: AR (Figure 2a) and callus (Figure 2b) formation.

Environmental modulation had little to no impact on the occurrence of either adventitious rooting or callus formation within the examined time points. In the summer, cuttings from the FR-treated easyR AEC0144 mini-stumps were less likely to root than the Ctrl ones. During the same season, the callus formation frequency was increased in the hardR 6201 SH and MT cuttings. In the fall, decreased callogenesis took place in the hardR 6201 FR cuttings.

Seasonality played a role in the AR and callus formation events. At the sampled timepoints, no ARs were observed in winter, at which time callus formation peaked for all the clones. At 21 dpe, the calli at the base of *E. dunnii* frequently had AR primordia-like protrusions under 1 mm in length that could explain the rooting rates measured around 30 dpe. The frequencies were largely uniform between the remaining seasons but varied across species in the same period. The EasyR AEC0144 cuttings were consistently more likely to develop ARs than the hardR *E. dunnii* clones. Callus-derived ARs were common in the hardR clones (Figure S2).

**Figure 3:**
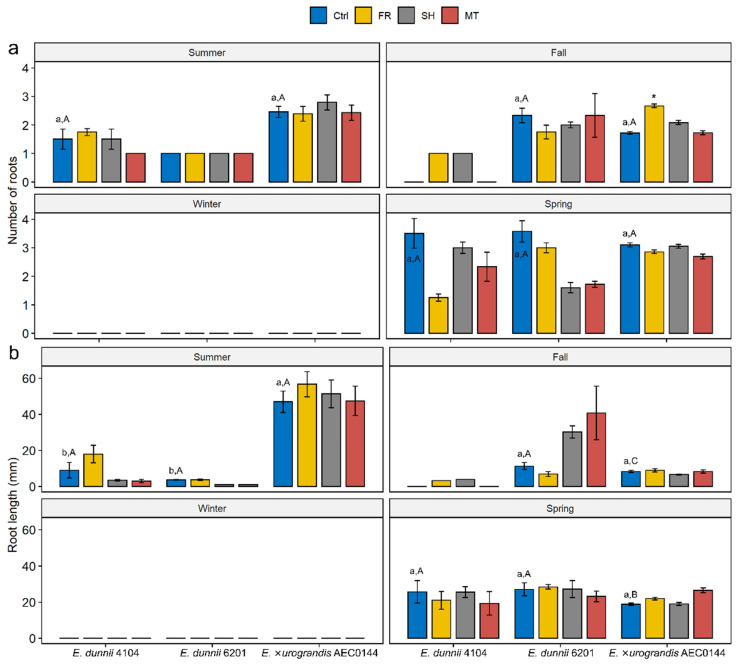
Effects of year-round mini-stump environmental modulation on the morphology of the adventitious root (AR) system in *Eucalyptus dunnii* 4104 and 6201 (hardR clones) and *E.* ×*urograndis* AEC0144 (easyR) cuttings at 21 and 14 days post-excision, respectively. (**a**) Number of ARs and (**b**) length (mm) of the longest AR in cuttings excised from mini-stumps under control (Ctrl), far-red-enriched (FR), shade nets (SH), and mini-tunnel (MT) conditions. Bars represent mean ± SEM. Asterisks indicate significant differences in treatments in comparison to the Ctrl (* *p* ≤ 0.05, Dunnett’s test). Different lowercase and uppercase letters indicate significant divergence when comparing the Ctrl results across clones in the same season and Ctrl results across seasons in the same clone, respectively (ANOVA, Tukey’s test, *p* ≤ 0.05).

When AR formation occurred, the root number (Figure 3a) and the length of the longest root (Figure 3b) were recorded at 14 and 21 dpe for the easyR and hardR clones, respectively. The callus size was found not to vary significantly (data not shown). The only instance of any treatment effect on the number or size of the ARs was measured in the fall for the easyR AEC0144 FR cuttings, which featured higher root number (Figure 3a). Similarly, no quantifiable significant variation was detected across seasons or species regarding the AR number. Nonetheless, seasonal changes in root length were found in the easyR AEC0144 cuttings, with a sharp decrease from summer to fall, followed by a partial recovery in spring. While broadly balanced between clones during most of the year, in the summer, root length was markedly greater in easyR AEC0144 than in the hardR clones.

### 2.3. Carbohydrate Content

For the more detailed analyses, *E. dunnii* 6201 and *E.* ×*urograndis* AEC0144 were chosen as representatives of the hardR and easyR phenotypes, respectively. Summer and winter were chosen as contrasting seasons for further comparisons based on the climate historic means of the subtropical region where this study was conducted, which showed higher differences in the photoperiod, temperature, and humidity between them (Table S1).

As an essential component of AR development, the levels of soluble sugars and starch were monitored after cutting excision in the hardR and easyR species during the contrasting seasons. The overall positive effect of the MT treatment in summer vs. its negative impact in winter was also taken in consideration when the seasons were narrowed down. These seasons showed contrasting environmental parameters and treatment effects. Only two of the three initial clones were analyzed due to the scarcity of hardR 4104 plant material and losses in the hardR 4104 mini-stump viability registered throughout the year.

**Figure 4:**
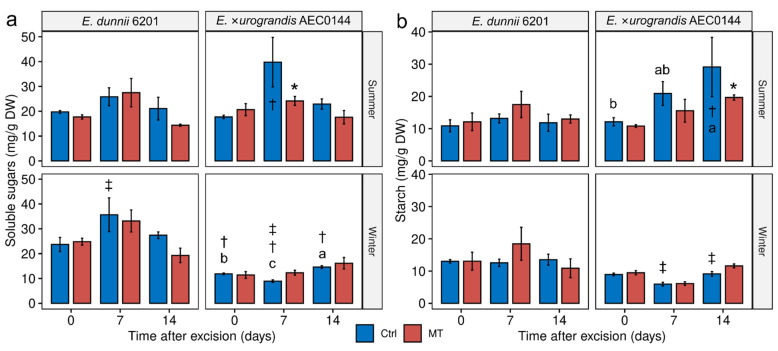
Carbohydrate concentration in *Eucalyptus dunnii* 6201 (hardR) and *E.* ×*urograndis* AEC0144 (easyR) cuttings in contrasting seasons. Whole-cutting (**a**) soluble sugar (mg/g DW) and (**b**) starch (mg/g DW) content at 0, 7, and, 14 days post-excision from mini-stumps under control (Ctrl) and mini-tunnel (MT) conditions. Bars represent mean ± SEM (*n* = 3). Asterisks (*), daggers (†), and double daggers (‡) indicate significant differences between the Ctrl and treatments, between clones in comparison to the Ctrl, and between seasons in comparison to the Ctrl, respectively. Different lowercase letters indicate Ctrl timewise variation (factorial ANOVA, estimated marginal means test, *p* ≤ 0.05). DW = dry weight.

Whole-cutting carbohydrate analysis revealed that the MT treatment had limited effects on the overall soluble sugar (Figure 4a) and starch (Figure 4b) levels. However, season- and species-related differences could be observed.

For instance, in the winter, the easyR AEC0144 soluble sugar levels were lower than those of hardR 6201 at all the sampled times. There was also significant timewise variation in these levels, with a transient decrease at 7 dpe. This timepoint coincided with the most inter-season variation, increasing from summer to winter in hardR 6201 and decreasing for easyR AEC0144.

The starch levels were similar between the species. Remarkably, the hardR 6201 starch levels were stable across the seasons, while the easyR clone displayed seasonal patterns. In the summer, its starch levels increased timewise, while in the winter, no such variation was detected. From 7 dpe, the starch content was lower than that observed in the summer.

### 2.4. Gene Expression Patterns

**Figure 5:**
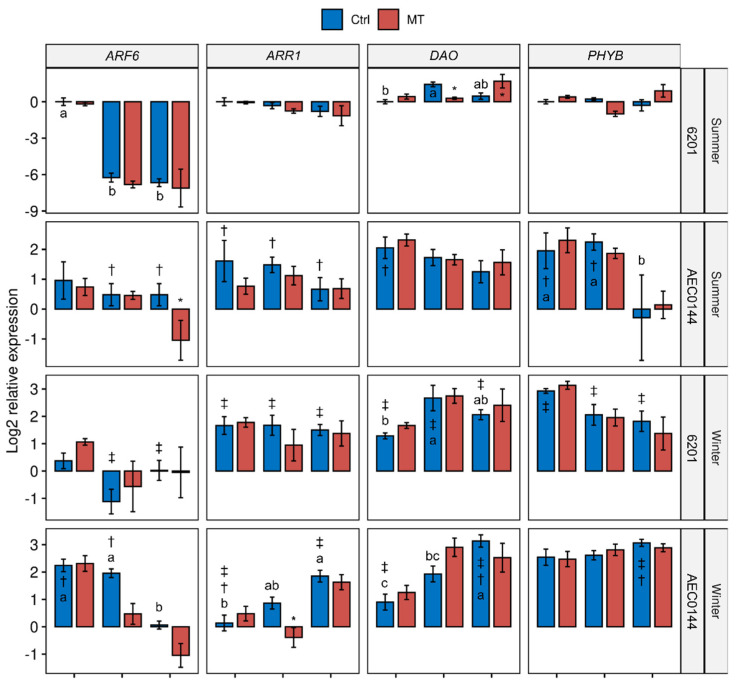

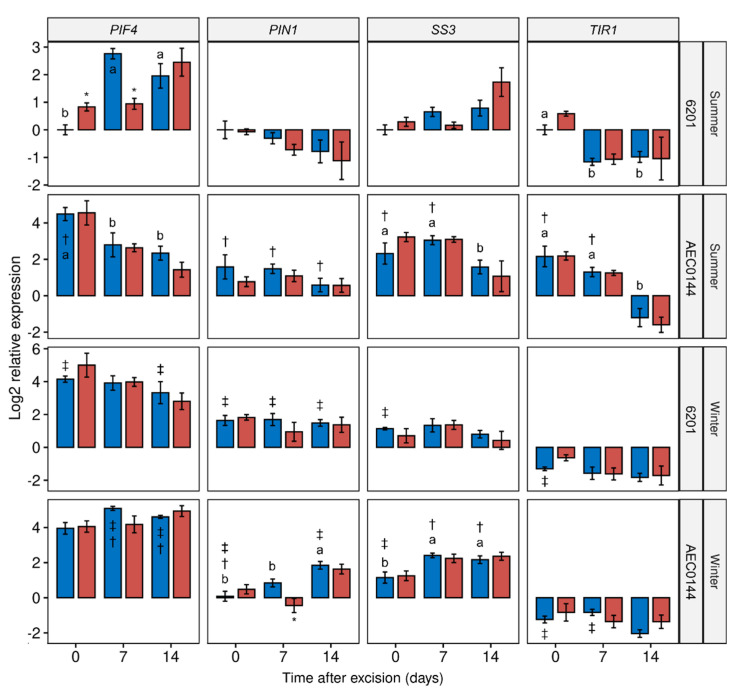
Transcriptional profiles of *E. dunnii* 6201 (hardR) and *E.* ×*urograndis* AEC0144 (easyR) cuttings. Log_2_-transformed RT-qPCR relative expression levels in whole cuttings at 0, 7, and 14 days post-excision from mini-stumps under control (Ctrl) and mini-tunnel (MT) conditions during summer and winter. Bars represent mean ± SEM (*n* = 3). Asterisks (*), daggers (†), and double daggers (‡) indicate significant differences between the Ctrl and treatments, between clones in comparison to the Ctrl, and between seasons in comparison to the Ctrl, respectively. Different lowercase letters indicate Ctrl timewise variation (factorial ANOVA, estimated marginal means test, *p* ≤ 0.05).

The transcriptional control of plant development plays a crucial role in adaptive growth processes such as pruning-induced axillary branching and cutting-induced adventitious rooting. The whole-plant gene expression levels (Figure 5) of propagation-relevant genes were measured in the hardR 6201 and easyR AEC0144 Ctrl and MT cuttings at different stages of development. Among the assessed genes were some whose expression levels had previously been shown [18,19,20] to correlate positively (*AUXIN RESPONSE FACTOR 6* (*ARF6*), *PIN-FORMED1* (*PIN1*), *TRANSPORT INHIBITOR RESPONSE 1* (*TIR1*)) and negatively (*ARABIDOPSIS TYPE-B RESPONSE REGULATOR 1* (*ARR1*)) with AR development. Additionally, genes associated with environmental cues, including *PHYTOCHROME INTERACTING FACTOR 4* (*PIF4*) and *PHYTOCHROME B* (*PHYB*), and auxin and starch metabolism, including *DIOXYGENASE FOR AUXIN OXIDATION* (*DAO*) and *STARCH SYNTHASE 3* (*SS3*), were also examined.

**Figure 6:**
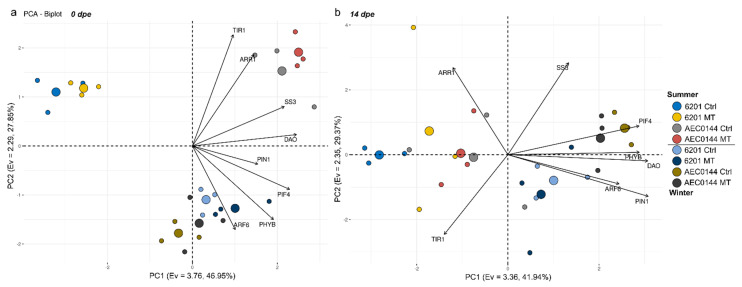
Principal component analysis of the relative gene expression levels at 0 dpe (**a**) and 14 dpe (**b**) in *E. dunnii* 6201 (hardR) and *E.* ×*urograndis* AEC0144 (easyR) cuttings. Axes represent principal components (PC) with the corresponding eigenvalues (Ev) and percentage of variance explained. Regular circles represent biological replicates (*n* = 3), while larger ones are the centroids for each group indicated in the legend.

Generally, the MT treatment did not significantly affect the expression patterns profiled in this study (Figure 5). This is reflected in the principal component analysis (PCA) results for 0, 7, and 14 dpe (Figure 6a, Figure S3 and 6b respectively). Interestingly, there seemed to be a greater treatment effect on the hardR 6201 cuttings at 7 and 14 dpe than at 0 dpe. This was evidenced by the larger distance between the hardR 6201 Ctrl and MT centroids on the PC1 × PC2 plane. Otherwise, the Ctrl–MT pairs tended to cluster together at both timepoints.

All the analyzed genes displayed timewise variation in their expression levels in at least one species × season combination. Where there was more than one case of temporal variation, these changes were, at times, uniformly crescent, as in *DAO*, or decrescent, as seen in *ARF6* and *TIR1*. For the other genes, there were mixed tendencies depending on the species and season. This was the case for *PIF4* and *SS3*.

In a comparison of the three timepoints, the overall expression patterns of the studied genes were closer between 0 and 7 dpe than 14 dpe in all cases (Figure 6a,b and Figure S3). Shifts could be seen both in the distribution of variables and in that of the data points across the sampled timepoints. For instance, *ARF6* expression appeared to have a transient shift in behavior at 7 dpe (Figure S3). From 0 to 14 dpe, the shifts were even larger, especially for *ARR1*, *TIR1*, and *SS3* (Figure 6). As for the data point displacement, a remarkable shift was seen in easyR AEC0144 but not in hardR 6201.

During the summer, at 14 dpe, it was common for the easyR AEC0144 cuttings to have developed ARs, while hardR 6201 had barely visible ARs or none. Even at 21 dpe, 6201 ARs were shorter than those of easyR AEC0144 at 14 dpe in summer (Figure 3b). Therefore, the overall gene expression patterns shifted more with time in the easyR clone than in hardR 6201.

Seasonality was a major factor affecting the measured expression levels regardless of the sampling time, albeit in different ways. Data from the different seasons were separated along the PC2 axis at 0 dpe (Figure 6a). Both the hardR 6201 and easyR AEC0144 cuttings obtained during winter were associated with higher levels of *DAO, PIF4*, *SS3*, and *PHYB* expression at 0 dpe, a pattern similar to that of easyR AEC0144 in summer. However, the former two differed from the latter due to their decreased *TIR1* and *ARR1* and increased *ARF6* expressions. The summer hardR 6201 cuttings markedly diverged from the other clusters, with lower expression levels of *PIF4*, *PHYB*, *DAO*, and *SS3*.

These three clusters showed little change at 7 dpe (Figure S3). At 14 dpe, this scenario changed (Figure 6b). The data from summer were all clustered together, while those from winter moved farther apart, in both cases due to the displacement of the easyR AEC0144 datapoints. The contrasting seasons formed two clusters along the PC1 axis. This means that a “summer” profile correlated with lower *PIN1, DAO, PHYB, PIF4*, and *ARF6* expression levels. The opposite is true for a “winter” profile. The distance between the AEC0144 and hardR 6201 samples along the PC2 axis was due to the higher expression levels of *SS3* in the former and lower levels of *ARR1* and *TIR1* in the latter.

### 2.5. Foliar Nutritional Profile

Adequate mineral nutrition is necessary for developmental processes such as mini-stump branching and AR formation in cuttings. Furthermore, the environmental modulation of mini-stump growth can interfere with nutrient acquisition. The leaf nutrient profiles of the Ctrl and MT-treated mini-stumps (0 dpe) and corresponding cuttings (7 dpe) were examined in the summer. This season was chosen because it was when the MT treatment increased the overall productivity of both hardR 6201 and easyR AEC0144.

**Figure 7:**
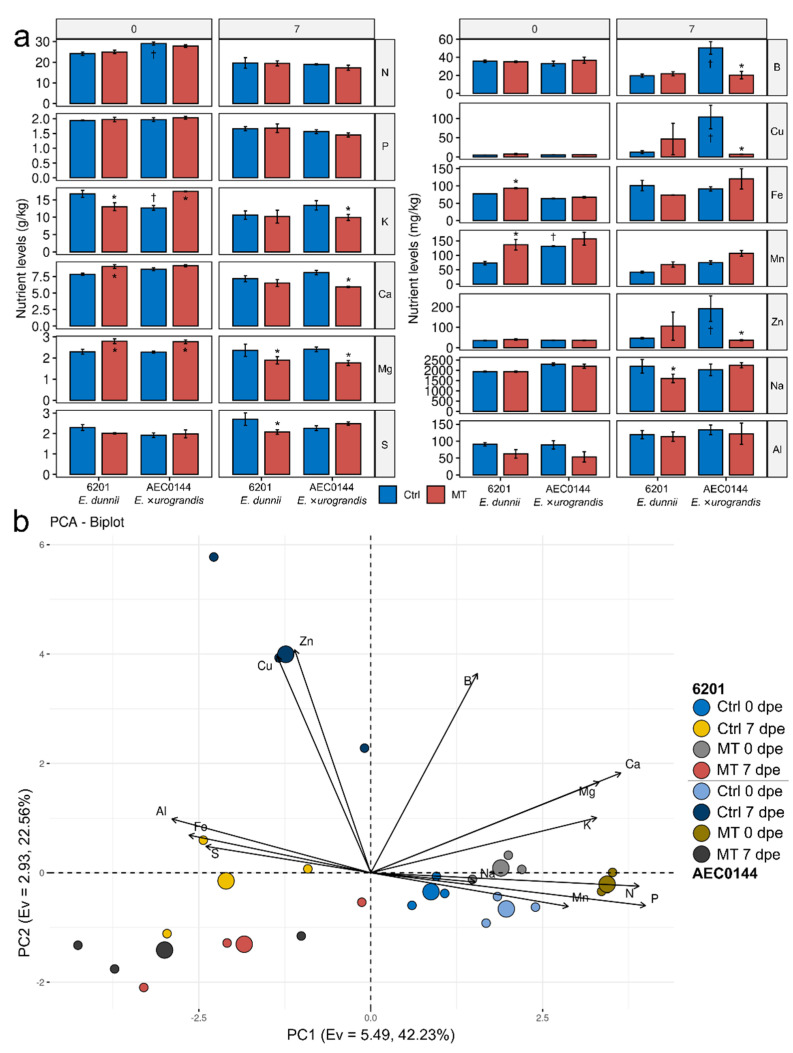
Foliar nutrient profiles of *Eucalyptus dunnii* 6201 (hardR) and *E.* ×*urograndis* AEC0144 (easyR) at the time of and post-cutting excision. **(a)** Levels of N, P, K, Ca, Mg, S (g/kg DW), B, Cu, Fe, Mn, Zn, Na, and Al (mg/kg DW) in the mini-stumps (0 days post excision, dpe) under control (Ctrl) and mini-tunnel (MT) conditions and respective cuttings (7 dpe). Bars represent mean ± SEM. Asterisks (*) and daggers (†) mark significantly different nutrient levels between treatments and species at each timepoint, respectively (factorial ANOVA, estimated marginal means test, *n* = 3–4, *p* ≤ 0.05). DW = dry weight. (**b**) Principal component analysis of foliar nutrient levels. Axes represent principal components (PC) with the corresponding eigenvalues (Ev) and percentage of variance explained. Regular circles represent biological replicates (*n* = 3), while larger ones are the centroids for each group indicated in the legend.

The foliar levels of some nutrients were significantly different between the MT-treated and Ctrl mini-stumps (Figure 7a). The MT-treated hardR 6201 mini-stumps (0 dpe) had increased levels of Ca, Mg, Mn, and Fe and decreased K content. The easyR AEC0144 mini-stump foliar nutrient profile was less impacted, with gains limited to the K and Mg levels, but with no losses. Due to the fundamentally different fertilization regimes of mini-stumps and cuttings, the associated nutrient levels were not compared across timepoints. Interestingly, while the MT treatment effect was largely linked to increases in the mini-stump foliar nutrient concentration, at 7 dpe, the opposite was observed. The hardR 6201 MT cuttings (7 dpe) had lower Mg, S, and Na contents than the Ctrl ones. The easyR AEC0144 MT cuttings were more impacted, with decreases in K, Ca, Mg, B, Cu, and Zn levels.

Nutrient analysis also revealed a few differences between the two clones. The easyR AEC0144 Ctrl mini-stumps (0 dpe) had higher N and Mn and lower K contents than hardR 6201. At the cutting stage (7 dpe), differences were limited to B, Cu, and Zn, which were found in higher concentrations in easyR AEC0144. A multivariate approach to PCA illustrated the relative position of each datapoint in the nutrient profiling dataset (Figure 7b). Together, the first three PCs accounted for 75.21% of the explained variance. PC1 correlated positively with the P, N, Ca, Mg, K, and Mn levels and negatively with those of S, Fe, and Al. Most of the data fell along the PC1 axis, except for those of the easyR AEC0144 MT cuttings at 7 dpe. Situated at the far positive side of PC2, this reflected the strong correlation between the vertical axis and the Zn, Cu, and B concentrations. PC3 (eigenvalue = 1.35, 10.41% of the explained variance, not featured) correlated with the Na and S levels.

The distance between Ctrl–MT pairs indicates the impact of the treatment on cutting foliar nutritional status. At 0 dpe, an MT-associated rightward shift from the Ctrl could be seen in both clones. Similarly, an MT downward displacement from the Ctrl was visible at 7 dpe. The baseline fertilization was different at 0 and 7 dpe, and the data from each timepoint were grouped on each side of the PC1 axis. The grouping of the 0 dpe data was more cohesive than that of the 7 dpe data, which displayed varying but consistent levels of leftward displacement from the former. In the 7 dpe scattered group, confined to the PC1 < 0 quadrants, the easyR AEC0144 Ctrl–MT results appeared to be further apart than those of hardR 6201. These data indicate that MT was able to modulate mineral nutrition of leaves relative to Ctrl and that variations in the nutrient concentration were affected by time.

## 3. Discussion

### 3.1. Mini-Tunnels Modulate Overall Productivity in a Season-Dependent Manner by Increasing Branching Rates

This study examined the effects of environmental modulation on commercial *Eucalyptus* propagation by cuttings, considering the productivity of mini-stumps as an objective parameter of the mini-garden performance. The light modulation of AR development, in terms of both irradiance and quality, has produced varying results according to different genetic backgrounds [24]. In the present study, mini-stumps were subjected to treatments that lowered the overall irradiance (SH) and that enriched the received spectrum in the FR region (FR). These treatments were generally detrimental to branching, even though, at times, they had positive impacts on rooting. This may be partly explained by the induction of shade-avoidance-response (SAR)-like effects by the reduction in the photosynthetically active light flux and lower R:FR ratios [31,32]. These results do not corroborate previous findings on in vitro *E. globulus* micro-cuttings, which benefited from FR enrichment [18]. The light modulation of commercial-level *Eucalyptus* propagation requires dedicated studies to achieve an optimal treatment duration (e.g., period of the day, length of treatment application) so that the side effects do not outweigh the potential benefits. A more recent work found that *E. dunnii* mini-stump branching rates could be enhanced by exposure to red (R) and FR LEDs under MTs [33]. This investigation differs from the present study in that its authors supplemented the mini-stumps with R light as well as FR light for 14 h a day, whereas herein, the FR treatment filtered out nearly all the R light and lasted for about half the time.

During winter, when FR and SH modulated the rooting in a clone-dependent manner, there was no associated loss in the branching rates. The beneficial effects of irradiance-reducing treatments in winter may also be related to protection against photoinhibition under low temperatures, particularly in the morning [34]. In *Petunia* cuttings, dark exposure is known to enhance adventitious rooting, even more so at lower temperatures. The light control of cutting development is also closely related to carbohydrate dynamics [35,36].

The use of MTs led to gains in the productivity of all three clones in the summer due to their enhanced branching rates. During the winter, it negatively impacted cutting productivity of the hardR clones through a significant reduction in rooting rates. This indicates a season- and clone-dependent mechanism that can affect the branching and rooting rates, two of the main components of *Eucalyptus* propagation. It has been reported that MT-treated mini-stumps have higher branching rates, particularly in warmer seasons, and that MTs impact branching more than rooting [30].

The effect of MT use on the rooting rates of four *Eucalyptus* clones at the mini-stump and cutting stages were analyzed [29]. Similar to the findings of this study, the MT effects varied across clones, and seasonality played an important role. MT treatment had a positive effect on the summer rooting rates for three out of the four clones, ranging from hardR to easyR. The same study showed no significant change in the air temperature apart from a decrease in the amplitude of the temperature variation. It was also shown that MTs can maintain high relative humidity levels for longer periods and cause some level of CO_2_ entrapment [29]. A strong genotype-linked effect has been reported for the rooting of carnation cuttings [37].

### 3.2. Seasonality and Species Are Major Factors in Cutting Developmental Outcomes

Callus formation was more frequent in the hardR clones. Callogenesis peaked in winter, when the rooting rates at 30 dpe were comparable to those in summer. However, no ARs were recorded at 14 and 21 dpe in the easyR AEC0144 and hardR *E. dunnii* cuttings, respectively. This indicates that, in winter, rooting took place after these sampling times, and that for the majority of the hardR *E. dunnii* cuttings, these were callus-derived ARs. Callus-derived ARs have been reported as relatively weakand more likely to cause young trees to fall [38]. While the treatments did not have a strong impact on the frequency of callogenesis, its more frequent occurrence in the hardR 6201 MT cuttings in the summer is in line with findings for MT-treated *E. benthamii* cuttings. The same study also recorded the lowest rooting performance coinciding with the highest callogenesis levels [28].

Although there is no single explanation as to what determines whether a cutting will develop ARs directly or indirectly, some features of plant propagation have been associated with more frequent callogenesis, and a recent study examined anatomical differences between calli and ARs [39]. Woody species are known to gradually lose their adventitious rooting ability with ontogenetic aging [19,40,41] and are more likely to undergo callus formation.

AR development monitoring was performed by recording the number of roots and the length of the longest AR. In the summer, the only season in which there was clear variation between the clones, easyR AEC0144 had more abundant and longer roots than the hardR *E. dunnii*, despite having been in the rooting greenhouse for one less week. This confirms the expected phenotype of the easyR clone. The lack of consistentand significant variation may be due to the small number of adventitious rooting events in the hardR *E. dunnii* cuttings and the large variation in form among the AR systems developed.

### 3.3. Global Carbohydrate Content Is more Sensitive to Seasonality and Species Effects Than to Mini-Tunnel Treatment

The establishment of the cutting base as a sink is of the most important steps leading to AR development [10,11]. HardR 6201 maintained a similar variation profile in its soluble sugar content in summer and winter. Conversely, different patterns were observed across the seasons in the easyR clone. The hardR 6201 cuttings had higher soluble sugar levels when compared to the easyR AEC0144 ones in winter. This may be related to the former’s frost tolerance since soluble sugars may act as cell-compatible osmolytes and help to avoid freezing [42]. In fact, there are reports of higher soluble sugar concentrations in frost-tolerant *E. dunnii* when compared to non-tolerant *E. grandis* [43].

Although, in winter, the hardR 6201 cuttings’ rooting rate was negatively affected by the MT treatment, no significant changes were observed in the MT carbohydrate content at the sampled timepoints. While the 30 dpe rooting rates remained the same for easyR AEC0144 in winter, the absence of formed roots by 14 dpe coincided with lower 7 dpe soluble sugar levels when compared to summer. The easyR AEC0144 cutting starch levels were also lower at 7 dpe, which may have impacted rooting speed. EasyR *E.* ×*urograndis* is a hybrid that is mostly cultivated in tropical regions and is likely to be more impacted by seasonality than subtropical species [44] such as hardR *E. dunnii*.

### 3.4. Contrasting Seasons Are Linked to Consistently Different Cutting Expression Profiles of AR-Related Genes

AR development is regulated by a crosslinked network of hormone signaling, which affects developmental processes through the transcriptional reprogramming of the target cells [45]. Our analysis of the gene expression at 0 dpe, i.e., at the time of excision, revealed different patterns between the summer and winter mini-stumps. The former correlated with higher levels of *TIR1*, *ARR1*, and *ARF6* expression, while the opposite was observed in the latter. Fewer ARs developed in Arabidopsis *tir1-1* mutants [16], and the localized expression of *TIR1* in pre-etiolated flooded WT seedlings was also reported [17]. For both clones, *TIR1* expression followed a decreasing trend after cutting excision. This is line with the current auxin signaling model for *Petunia* cuttings, wherein a period of low auxin sensitivity follows auxin maximization at the cutting base [10,46]. The drop in *TIR1* expression was, however, more gradual in the easyR cuttings than in those of 6201. This may point to a longer window of auxin sensitivity that allowed for more AR induction events to occur, leading to a greater number of ARs. During the winter, the *TIR1* expression levels were low from the start.

The *ARF6* expression sharply decreased between 0 and 7 dpe in the hardR 6201 cuttings in the summer. A similar decrease was detected between 7 and 14 dpe in the easyR AEC0144 cuttings in the winter. As has been hypothesized for *TIR1*, *ARF6* expression may decrease once the effects of transient auxin accumulation have subsided. Nonetheless, stable levels of *ARF6* expression were found up to 8 dpe in *E. globulus* and *E. grandis* micro-cuttings grown in vitro [20]. A similar study reported an increase in *ARF6* expression of *E. globulus* micro-cutting at 9 dpe, well into the AR formation stage for the model used [18]. For the cuttings used in the present study, however, this phase was reached at 14 dpe by easyR AEC0144 and even later by the hardR 6201 cuttings. No such increase was seen in any of the clones. *PtRR13*, the *Populus* homolog of *Eucalyptus ARR1*, is a negative regulator of AR formation [47]. In the present study, however, higher *ARR1* expression was observed in the easyR clone during the summer. These different profiles may reflect the very distinct cultivation conditions, plant ages, rooting phases, and evaluation strategies.

While, in winter, the gene expression profiles at 0 dpe of both clones were similar, in summer, the opposite was observed. The easyR AEC0144 cuttings were associated with higher *DAO*, *PIF4*, *SS3*, *PHYB*, and *PIN1* expression levels, while the opposite was seen in the hardR 6201 cuttings.

Auxin catabolism helps to fine-tune the spatiotemporal organization of developmental processes. Interference with the *DAO* apple homolog *ADVENTITIOUS ROOTING-RELATED 1* has been shown to enhance rooting competence [48]. Arabidopsis *dao1-1* mutants developed more ARs than WT seedlings [49]. The temporal variation in the *DAO* expression levels was almost uniform between the clones and seasons, with a crescent tendency. The proposed mechanism of DAO’s influence on adventitious rooting is based on the Arabidopsis etiolated seedling model and involves the inhibitory effect of jasmonate application. In cuttings, the role of jasmonate is not that of an inhibitor [50]. More detailed mechanistic studies on the role of DAO in wound-induced AR development must be performed to verify the extent to which this translates to cuttings.

In Arabidopsis, PHYB enhances branching by impairing auxin signaling [51,52]. In the summer, the higher *PHYB* expression at 0 dpe in easyR AEC0144 coincided with its better branching performance. However, equally high expression levels in winter were associated with low branching results. Arabidopsis *phyB* mutants, under low irradiance levels, produced more ARs, revealing a possible link between PHYB, light intensity, and AR development [53]. PIF4 is part of the branching suppression mechanism that ensues in the absence of active PHYB in Arabidopsis [54]. The *PIF4* expression levels were elevated in both clones in winter but were higher in the clone with the best rooting performance. The PHYB-PIF signaling module relies heavily on post-translational modifications, especially phosphorylation [54,55], which occur downstream of any of the transcriptional program changes measured herein. The significant increase in the *PIF4* expression levels at 0 dpe in the hardR 6201 MT cuttings corroborates the reports of higher expressions linked to high temperatures in Arabidopsis [56]. This effect was not seen in easyR AEC0144 in the summer or any of the clones during the winter. The effect of seasonality on the diel *PIF4* expression levels has been reported in Arabidopsis, and warmer seasons with longer days were linked to higher transcript levels [57].

*SS3* expression was higher in the easyR AEC0144 cuttings than in the hardR 6201 ones at the timepoints in both the seasons. In easyR AEC0144, the summer *SS3* expression peaked at 7 dpe, a week before the highest levels of starch were measured for this clone, when its expression levels decreased. This finding might illustrate the delay between transcriptional reprogramming and the metabolic effect. Albeit at a lower level than in the warm season, during winter, the easyR AEC0144 *SS3* expression increased from 7 dpe onwards and did not translate into a higher starch content a week later. A transient increase in the *SS3* expression was observed during the in vitro adventitious rooting of the hardR *E. globulus* micro-cuttings under far-red-enriched light, associated with higher rooting rates [18].

PIN1 involvement in AR development was first shown in rice [58]. The overall higher *PIN1* expression coincided with easyR AEC0144’s better performance than hardR 6201 in the summer. The *PIN1* expression levels also increased in winter, albeit not to the same extent. PIN1 is also a target of strigolactone action in the modulation of shoot branching [59]. Since only whole-cutting samples were analyzed, differences in the shoot apex vs. base are unknown. Therefore, direct correlations between the expression data and organ-specific growth could be misleading. Transient increases in *PIN1* expression have been measured in *E. globulus* micro-stumps and micro-cuttings grown in vitro during the induction phase [18,20].

Through multivariate analysis, distinct trends in gene expression could be observed in the hardR and easyR clones. Displacements in the datapoint distribution, although not causally linked to any particular biological phenomenon, can show whether the two clones undergo similar changes in the expression levels from one timepoint to the next. Shifts along the two axes must be interpreted by considering the correlation coefficients of each variable with PC1 and PC2 (fully available in Tables S3–S6). These patterns are, however, contingent to the set of assessed parameters. As such, though their interpretation may provide insights into the overall shape of the datasets that can hint at the variables of interest, they do not provide causal evidence for mechanistic model construction alone.

Between 0 and 14 dpe, a significant shift in the expression profiles was observed. The expression data of the summer cuttings correlated negatively with *PIN1*, *DAO*, *PHYB*, *PIF4*, and *ARF6*. The inverse was seen in the winter samples. This temporal shift was recorded for easyR AEC0144 but not for hardR 6201, which may indicate the former’s ability to reprogram AR-related gene expression more effectively. It is also indicative of a less auxin-sensitive stage, in which AR induction has been completed, and further stages, such as elongation and emergence, have started. Among the winter datapoints, the AEC0144 cuttings were associated with higher expression levels of *SS3* and *ARR1* and lower *TIR1* expression. This feature of the easyR AEC0144 cutting gene expression profiles coincided with the superior productivity results of this clone and sheds light on its more rapid rooting response.

In this study, gene expression assays were performed using whole cuttings as the starting material due to logistical constraints. As a result, while these profiles are helpful in illustrating major differences between species, seasons, and timepoints, they fall short of revealing tissue-specific differences in gene expression. This exploratory analysis, despite being limited in resolution, was able to unveil patterns that are worth exploring. Further directed studies examining basal stem cambial gene expression versus that of the upper shoots will be able to dissect these patterns and provide a more complete picture of the gene expression profiles of hardR and easyR species.

### 3.5. Mini-Tunnels Significantly Alter Leaf Nutrient Profiles of Mini-Stumps and Cuttings in the Summer

The MT-treated mini-stumps and cuttings were found to have different foliar nutrient profiles. At 0 dpe, although the significant clone-specific effects varied, the main affected nutrients were K, Ca, Mg, Fe, and Mn. Previous results of *E.* ×*urograndis* mini-stumps showed a negative correlation between the rooting rates and K and Ca levels. The same study revealed a positive correlation between the Mg, Zn, Cu, Mn, and B levels and rooting [60]. In addition to treatment-derived effects, the genetic background was found to be associated with different levels of some nutrients, notably N, K, and Mn. Increased foliar levels of N were measured in AEC0144 mini-stumps (0 dpe), the clone with the best propagation performance, in accordance with similar effects of N-enriched nutrition on *E. globulus* branching and rooting rates [61]. The same study revealed that K enrichment did not result in productivity gains. Mini-stump nutrition is known to influence rooting performance. In *E. benthamii*, increased Zn and B concentrations in the nutritive solution given to mini-stumps led to the best rooting performance in the cuttings [26]. This is in line with findings from in vitro propagation studies [25,62], pointing to Mn and Cu as positive regulators of AR development.

The excision of cuttings from mini-stumps is known to cause losses of minerals. In this study, after excision, the cuttings were transferred to a new fertilization regime. At 7 dpe, a week after this change in the baseline nutrition, the nutrient profiles were much less cohesive between the conditions (Ctrl and MT) and clones. Yet, it was still possible to identify MT-associated changes in the cutting nutrient profiles. The K, Ca, Mg, S, B, Cu, Zn, and Na levels were affected. The treatment effect on the global profile, however, was larger in the easyR AEC0144 cuttings. All the altered nutrients registered a decrease in their foliar concentration. The easyR AEC0144 Ctrl cuttings had higher levels of B, Zn, and Cu than the MT cuttings and Ctrl mini-stumps. This points to either a remobilization of nutrients from the nonfoliar tissues to the leaves and/or a more competent uptake of these nutrients from the substrate. Given the naturally heterogenous character of the nutrient distribution in the substrate, more biological replicates are needed to establish the reasons behind these differences. The hardR 6201 MT cuttings also had uniformly lower levels of all the altered nutrients, though these did not include B or Zn, but rather K, Ca, and Mg. A study on *E. cloeziana* propagation found a positive correlation between the rooting rates and cutting N, P, K, and B contents [63]. A similar study found *E. dunnii* rooting rates to be positively correlated with the P, K, B, and Na levels in cuttings [44].

Although no direct comparisons were drawn between 0 and 7 dpe, the distance on the PC1xPC2 plane between the two data groups can indicate a negative correlation with N, P, K, Ca, Mg, and Mn (Table S6). This may reflect losses associated with cutting excision. A study on *Corymbia citriodora* nutrient partitioning showed that potential cuttings (young branches) had higher concentrations of the more mobile nutrients, such as N, K, and S. In contrast, B and less mobile nutrients, such as Ca and Zn, were confined to the lower region of the mini-stump shoots and stems. Most of the mini-stumps’ Al, Fe, and Na contents were restricted to the roots [64].

Some of the metabolic, transcriptional, and nutritional levels of regulation underlying distinct clonal propagation performances in environmentally managed *Eucalyptus* spp. clonal gardens were described herein. Both species and seasonal impacts were examined. The positive effects of MT use on the overall productivity were largely limited to the summer and were due to higher branching rates, whereas the effects of light modulation were less relevant and often negative in the experimental setup used. EasyR AEC0144, an *E.* ×*urograndis* clone, was most affected by environmental treatments, responding to much of the season- and time-associated variation in the parameters examined. EasyR AEC0144’s rooting performance was superior to that of the hardR 6201 *E. dunnii* clone throughout the year. The easyR clone also had a unique timewise shift in its expression profiles of the monitored genes, which was generally associated with a progressively lower auxin sensitivity. This pattern coincided with its better propagation performance during summer, perhaps due to seasonal changes in the auxin dynamics in both the mini-stumps and mini-cuttings. The largest variation in the foliar nutrient profile was seen in the cuttings (7 dpe), while at 0 dpe, the nutritional status was similar between the different species. MT use altered the foliar nutrient profiles at both timepoints and in both species.

These patterns contribute to the refinement of existing hypotheses on the phenomena of AR recalcitrance and seasonal influences on propagation in commercial clonal gardens. In association with future and ongoing studies, our findings may help to establish novel management strategies for the improved clonal propagation of *Eucalyptus*.

## 4. Materials and Methods

### 4.1. Plant Material and Study Site

Three eucalypt clones were used for the cutting productivity studies: *Eucalyptus dunnii* Maiden 4104 and 6201 and *E. urophylla* S.T.Blake × *E. grandis* W.Hill (hereafter termed *E.* ×*urograndis*) AEC0144. These clones were already in use at the Tecnoplanta Florestal nursery (Barra do Ribeiro, RS, Brazil). Whereas *E.* ×*urograndis* AEC0144 is an easy-to-root clone, both the *E. dunnii* clones are recalcitrant to adventitious rooting.

The mini-stumps were maintained in sand beds (Figure S4a) under drip fertigation consisting of (g/m^3^): Ca(NO_3_)_2_, 670.1; (NH₄)H₂PO₄, 476.1; KNO_3_, 274.4; K_2_SO_4_, 14.44; MgSO_4_, 347.3; CuSO_4_, 1.2; ZnSO_4_, 3.0; Na_2_MoO_4_, 0.8; and (cm^3^/m^3^) organic boron, 10.0. The cuttings were conditioned in plastic tubes pre-filled with a peat- and perlite-based substrate (Carolina Soil, Santa Cruz do Sul, Brazil) and slow-release fertilizers: (kg/m^3^ substrate) single super phosphate, 3.0; Osmocote^®^ 19-06-10, 1.5; and NPK 10-14-10, 3.0 (Yara, Brazil).

Study site meteorological data (air temperature, relative humidity, and day length) of 2019 were obtained from the Brazilian National Institute of Meteorology and are available in Table S1. The local climate is classified as Cfa according to the Köppen climate classification system.

### 4.2. Experiment Design and Treatments

One whole sand bed (*ca.* 20 × 5 m, L × W) was used for each clone. The lengthwise outermost portions were not used to avoid discrepancies. The remaining space was used to place 12 experimental units in total (4 conditions, *n* = 3) in a uniformly distanced manner. To standardize the treatment application, 36 hollow structures (60 × 50 × 50 cm, L × W × H) were fashioned out of PVC tubing and placed over each 300 cm^2^ experimental unit that would be subjected to each condition (Figure S5).

The control (Ctrl) mini-stumps were kept under a hollow structure without any additional features. For the far-red-enriched treatment (FR), the top face of the structures was covered with a plastic light filter (Supergel #91 Primary Green, Rosco, Brazil). For the treatment with reduced irradiance (SH), the top face of the structures was covered with a double layer of black plastic horticultural shade nets to ensure 50% less irradiance. Mini-tunnels (MT) were constructed by covering all but the bottom face of the structures with a 150 µm-thick layer of a transparent horticultural plastic cover (Figure S5).

The transmittance spectra of the materials used to fashion the FR and SH structures were verified using a field spectroradiometer (ADS Fieldspec 4, Malvern Pananalytical, Malvern, UK) and used to estimate the R:FR (red:far-red) ratios. The direct irradiance was halved by having the light pass through the material used to fashion the SH structures, with no detected selection of a particular wavelength, resulting in an R:FR ratio close to 1.0. The light filter used in the FR structures selectively filtered out nearly all the red, reducing the R:FR ratio to less than 0.1. Technical details of the FR filter are available in Figure S6.

The air temperature and relative humidity were measured from 9–11 am using a thermohydrometer (AK28, AKSO, São Leopoldo, Brazil). While in the summer an upward trend in the relative humidity was seen, no quantifiable significant changes in either parameter were observed in the MT structures, probably due to the time of day of the measurements and insufficient precision of the equipment. However, condensation was frequently observed inside the MTs in the morning, pointing to the possibility of a more robust change in the evening.

These structures, modified according to each treatment, were placed over each experimental unit daily at 4 pm local time and removed by 10 am on the following day. The same experimental units were used from January 2019 to January 2020. A summary of the experimental design and the analyses performed is available in Figure S7.

### 4.3. Cutting Excision and Rooting

Weekly, cuttings were excised from the mini-stumps inside the experimental units. Every qualifying branch, i.e., those 6–10 cm in length and containing two pairs of leaves, was cut. For each experimental unit, the total weekly number of cuttings obtained per mini-stump was recorded. These cuttings were immediately placed in plastic tubes pre-filled with rooting substrate and transferred to rooting greenhouses in large trays (Figure S4b), grouped according to the clone and treatment.

Thirty days after excision, the cuttings were assessed for rooting. The criterion used for the rooting assessment was a cutting’s ability to produce roots that ran along the whole plastic tube length of approx. 12 cm, i.e., they were visibly emerging from the bottom of each tube (Figure S8). An average of 55 cuttings were assessed for every clone, treatment, and season combination.

Using the branching and rooting rates, a combined metric termed “rooted cuttings per stump per month” was established. Each weekly observation of the branching data was converted into its monthly equivalent and then multiplied by the respective mean rooting rate at 30 dpe. The standard errors of each measurement were accounted for in the final metric.

### 4.4. Sample Collection, Morphological Assessments, and Chlorophyll Measurements

During the rooting process, i.e., from cutting excision to the 30-day rooting assessment, samples were collected for further molecular and biochemical analyses. These consisted of four whole cuttings, which were thoroughly washed in distilled water and dried with paper towels and then placed in aluminum foil packets and immediately frozen in liquid nitrogen. The sample collection was performed at 0 (following excision), 7, 14, and 21 days post-excision (dpe). The cuttings were later finely ground in liquid nitrogen and kept at −80 °C for further analysis. Due to the generally faster development of AEC0144, its cuttings were not sampled beyond 14 dpe.

The adventitious root (AR) development was also monitored before the final rooting assessment. At 14 dpe (all clones) and 21 dpe (*E. dunnii* clones), the cuttings (*n* = 4–8) were removed from the rooting substrates, washed in distilled water, and dried with paper towels, and both the number of ARs and the length of the longest AR (hereafter, the root length) were recorded. The roots were measured using a digital caliper and only accounted for if they were 2 mm or longer and bearing a polar tip. The chlorophyll fluorescence was monitored using a nondestructive chlorophyll meter (SPAD-502Plus, Konica Minolta, Tokyo, Japan).

### 4.5. Carbohydrate Content Analysis

The whole-cutting samples (*n* = 3) from 6201 and AEC0144 collected during the summer and winter at 0, 7, and 14 dpe were subjected to soluble sugar extraction, as previously described [65]. Briefly, approx. 50 mg of finely ground frozen plant tissue was incubated with 0.5 mL of 80% ethanol at 80 °C for 15 min for soluble sugar extraction. The mixture was centrifuged at 13,000× *g* for 15 min, after which the supernatant phase was recovered. This procedure was repeated three times, and the combined supernatants (just short of 1.5 mL) were used for soluble sugar quantification, while the pellets were saved for starch hydrolysis.

Starch acid hydrolysis [66] was performed twice by resuspending the pellets in 0.25 mL distilled water and 0.32 mL of 52% perchloric acid and placing the samples in an ultrasonic bath for 15 min. After 15 min of centrifugation at 13,000× *g*, the supernatant phases from both extractions were combined and used for starch quantification.

Both the soluble sugars and starch were quantified using the phenol-sulfuric acid method [65,67]. In short, 0.5 mL of 5% phenol solution was added to 0.2 mL supernatant, followed by the addition of 1 mL concentrated sulfuric acid. After vortexing, the reaction tubes were kept in the dark for 10 min and then placed in an ice bath to stop the reaction. The soluble sugar concentration was determined via spectrophotometry (490 nm) against a D-glucose standard curve. The starch content was similarly determined against a D-glucose in 29.2% perchloric acid standard curve, using 0.9 as the stoichiometric conversion factor.

### 4.6. Gene Expression Analysis

Total RNA extraction was performed using the NucleoSpin RNA Plant Kit (Macherey-Nagel, Duren, Germany) according to the manufacturer’s instructions. Due to the lower RNA quality of the samples collected during the summer, an alternative lithium-chloride-based protocol was used [68]. The quality of all the samples was verified in 1% agarose gel, and the RNA content was estimated using a spectrophotometer (L-quant, Loccus, Cotia, Brazil). First-strand cDNA synthesis was performed using 1 μg of total RNA extracted from the whole cuttings, oligo-dT primers, and M-MLV reverse transcriptase (Invitrogen, Waltham, MA, USA). The final cDNA products were 50-fold diluted in RNAse-free distilled water prior to use in qPCR. The qPCR reactions were performed as previously described [20]. The primers were designed using the Primer-BLAST tool [69] based on the *E. grandis* genome. The full list of the primers used is available in Table S2.

Reference genes were determined by testing the most stable candidates from a set identified for the in vitro AR of *E. globulus* [70], and their stability was assessed using the R package ‘NormqPCR’ [71]. The chosen genes were *HISTONE 2B* (*H2B*) and *SAND-DOMAIN PROTEIN* (*SAND*). The Cq and efficiency values were calculated from the raw fluorescence values using the ‘qpcR’ package [72]. The relative expression levels were determined using the ‘pcr’ package [73].

### 4.7. Leaf Nutrient Profiling

Approx. 50 g of fresh cutting leaf biomass from 6201 and AEC0144 at 0 and 7 dpe, collected during summer, were sent to IBRA (Instituto Brasileiro de Análises—Sumaré—SP, Brazil) for leaf nutrient analysis. The K, Ca, Mg, Na, Fe, Cu, Mn, and Zn levels were obtained by atomic absorption spectroscopy. Colorimetric methods were used for B, P, and Al quantification, while the S, Cl, and N levels were determined, respectively, by barium sulfate turbidimetric analysis, silver nitrate titration, and an adapted Kjeldahl method [74].

### 4.8. Statistical Analysis

The quantitative data were checked for normality and then submitted to factorial ANOVA followed by estimated marginal means, Tukey’s, or Dunnett’s post-tests. The binomially distributed proportional data were arcsine-transformed and then submitted to means comparison tests using appropriately calculated binomial standard deviations. Unless stated otherwise, the data are presented as mean ± SEM. Descriptive and inferential statistics were obtained using the R v. 4.1.1 [75] base functions and package ‘rstatix’ [76]. The charts were generated using the package ‘ggpubr’ [77]. Full ANOVA tables are available in Tables S7–S10.

Principal component analysis (PCA) of the log_2_-transformed and standardized data was performed using the packages ‘FactoMineR’ [78] and ‘factoextra’ [79]. The Kaiser criterion (eigenvalue > 1) was used to determine the number of principal components analyzed. The full correlation results are available in Tables S3–S6.

## Data Availability

The original contributions presented in the study are included in the article/Supplementary Material, and further inquiries can be directed to the corresponding author.

## Supplementary Materials

The following supporting information can be downloaded at: https://www.mdpi.com/article/10.3390/plants11233281/s1, Figure S1: Chlorophyll content of *Eucalyptus dunnii* 6201, 4104 (hardR clones), and *E.* ×*urograndis* AEC0144 (easyR); Figure S2: *Eucalyptus* spp. cutting morphology 21 days post-excision; Figure S3: Principal component analysis of relative gene expression levels at 7 dpe; Figure S4: Growth environments used for *Eucalyptus* propagation; Figure S5: Mini-stump environmental modulation; Figure S6: Primary Green light filter technical data sheet; Figure S7: Overview of the propagation experimental design and analyses performed for whole-cutting samples; Figure S8: Rooted *Eucalyptus* cuttings on a rooting greenhouse table; Table S1: Meteorological data for the study site; Table S2: List of primers used for gene expression analysis; Table S3: Pearson’s correlation coefficient between gene expression levels at 0 dpe and principal components; Table S4: Pearson’s correlation coefficient between gene expression levels at 7 dpe and principal components; Table S5: Pearson’s correlation coefficient between gene expression levels at 14 dpe and principal components; Table S6: Pearson’s correlation coefficient between foliar nutrient levels and principal components; Table S7: Productivity data ANOVA table; Table S8: Carbohydrate content ANOVA table; Table S9: RT-qPCR data ANOVA table; Table S10: Nutrient profile ANOVA table.
