# Supplementary material for: Environmental Modulation of Mini-Clonal Gardens for Cutting Production and Propagation of Hard- and Easy-to-Root Eucalyptus spp
Source: Plants (Basel). 2022 Nov 29;11(23):3281. doi: 10.3390/plants11233281 (PMC9740115; doi:10.3390/plants11233281)
Supplement: Supplementary file 1 [file plants-11-03281-s001.zip › plants-2006316-supplementary.pdf]

plants

MDPI

Article

Environmental Modulation of Mini-Clonal Gardens for Cutting Production and Propagation of Hard- and Easy-to-Root *Eucalyptus* spp.

Johnatan Vilasboa [1,2,†], Cibele T. Da Costa [1,3,†] and Arthur G. Fett-Neto [1,2,3,*]

[1] Plant Physiology Laboratory, Federal University of Rio Grande do Sul, Porto Alegre, RS 91501-970, Brazil; johnatan.vilasboa@gmail.com (J.V.); cibeletesser@gmail.com (C.T.D.C.)
[2] Center for Biotechnology, Federal University of Rio Grande do Sul, Porto Alegre, RS 91501-970, Brazil
[3] Department of Botany, Federal University of Rio Grande do Sul, Porto Alegre, RS 91501-970, Brazil
* Correspondence: fett.neto@ufrgs.br
† These authors contributed equally to this work.

Citation: Vilasboa, J.; Da Costa, C.T.; Fett-Neto, A.G. Environmental Modulation of Mini-Clonal Gardens for Cutting Production and Propagation of Hard- and Easy-to-Root *Eucalyptus* spp. *Plants* **2022**, *11*, 3281. https://doi.org/10.3390/plants11233281

Academic Editor: Uwe Druege

Received: José Manuel Pérez Pérez
Accepted: 18 October 2022
Published: 29 November 2022

Publisher's Note: MDPI stays neutral with regard to jurisdictional claims in published maps and institutional affiliations.

Copyright: © 2022 by the authors. Licensee MDPI, Basel, Switzerland. This article is an open access article distributed under the terms and conditions of the Creative Commons Attribution (CC BY) license (https://creativecommons.org/licenses/by/4.0/).

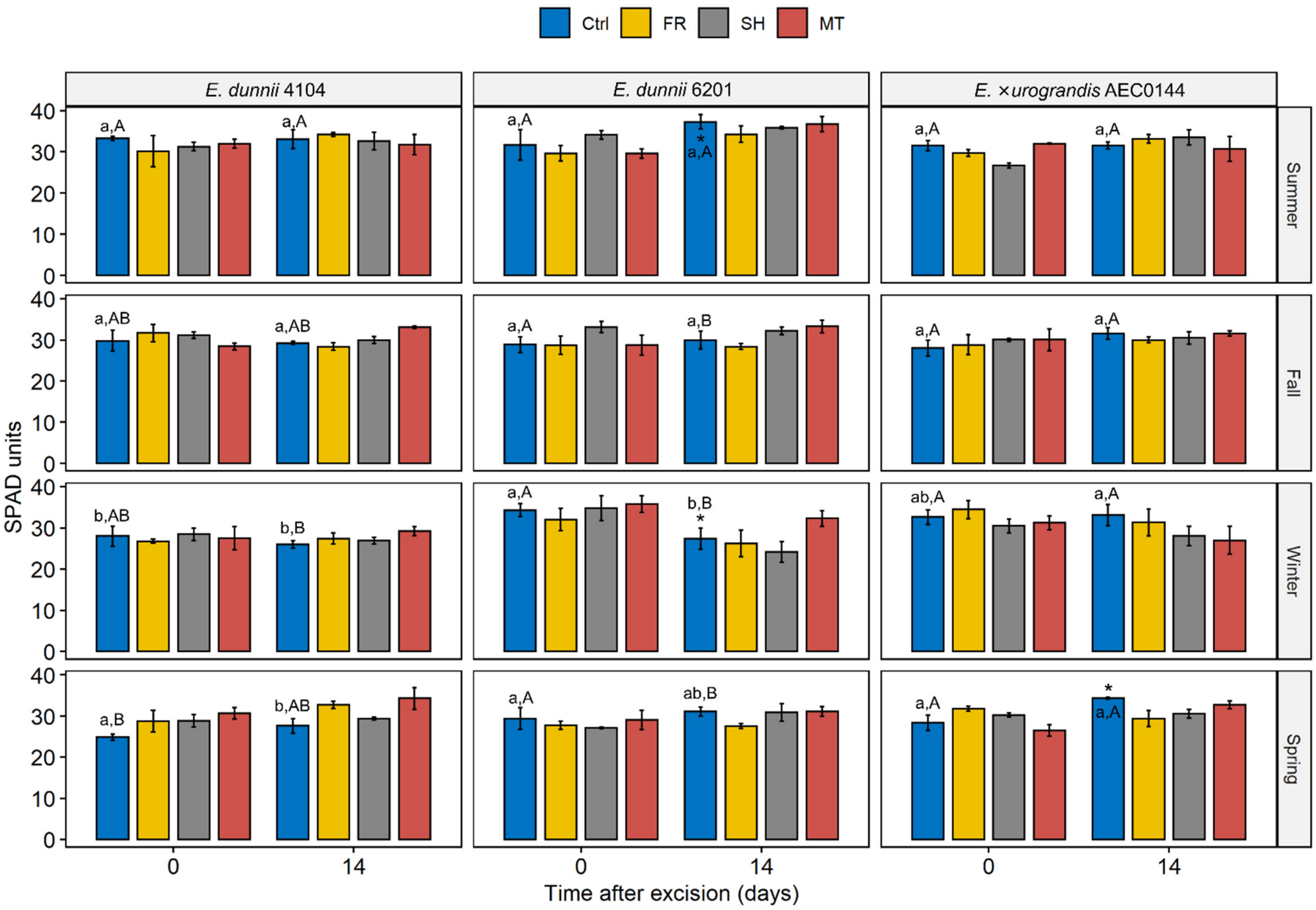

Figure S1. Chlorophyll content of *Eucalyptus dunnii* 6201, 4104 (hardR clones), and *E. ×urograndis* AEC0144 (easyR). Nondestructive chlorophyll measurements (SPAD units) of cuttings (n = 5) collected during summer, fall, winter, and spring under control (Ctrl), far-red enriched (FR), shade nets (SH), and mini-tunnel (MT) conditions. Bars represent mean ± SEM. Asterisks indicate significant difference between Ctrl cuttings at different timepoints (*p ≤ 0.05, Student's *t*-test). Different lowercase and uppercase letters indicate statistical significance when comparing Ctrl results across clones in the same season, and Ctrl results across seasons in the same clone, respectively, at the same timepoint (Factorial ANOVA, Estimated Marginal Means test, p ≤ 0.05).

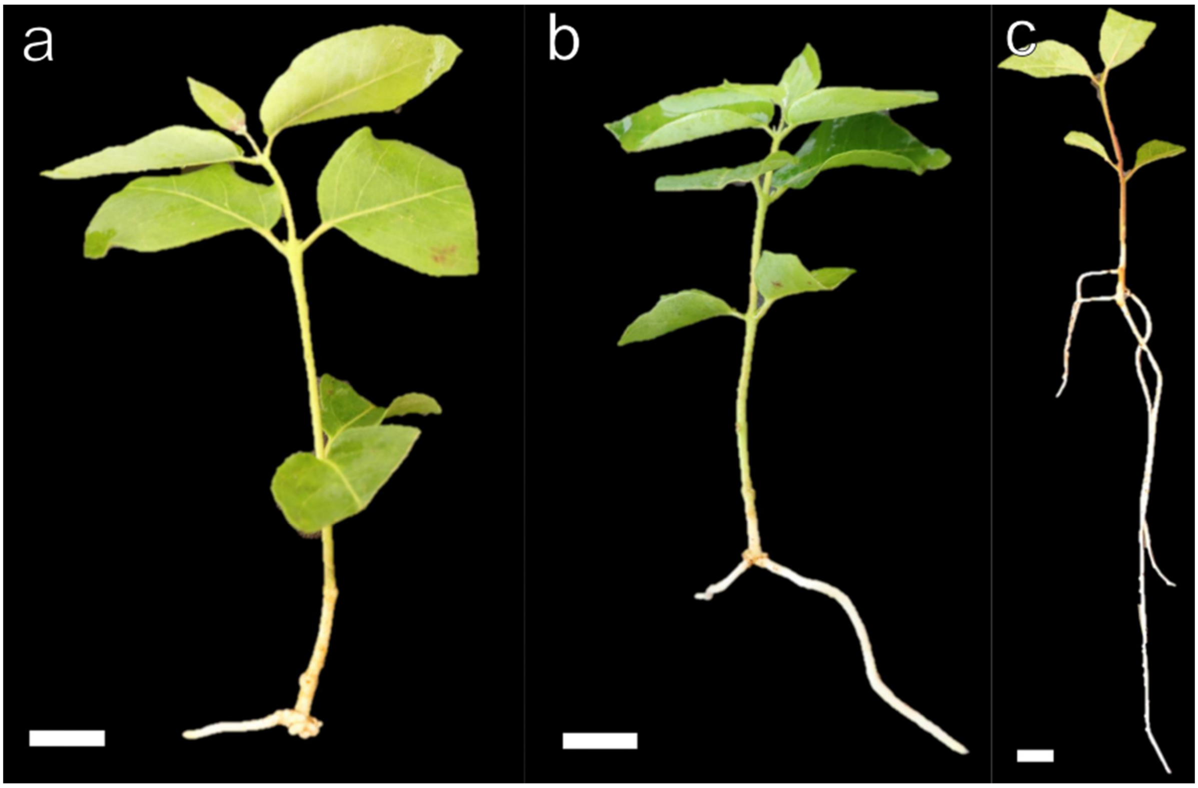

Figure S2. *Eucalyptus* spp. cutting morphology 21 days post excision. (a) *E. dunnii* 4104 and (b) *E. dunnii* 6201 (hardR) cuttings with callus-derived adventitious roots. (c) *E.* ×*urograndis* AEC0144 (easyR) with more pronounced, directly formed adventitious roots. Scale bar = 1 cm.

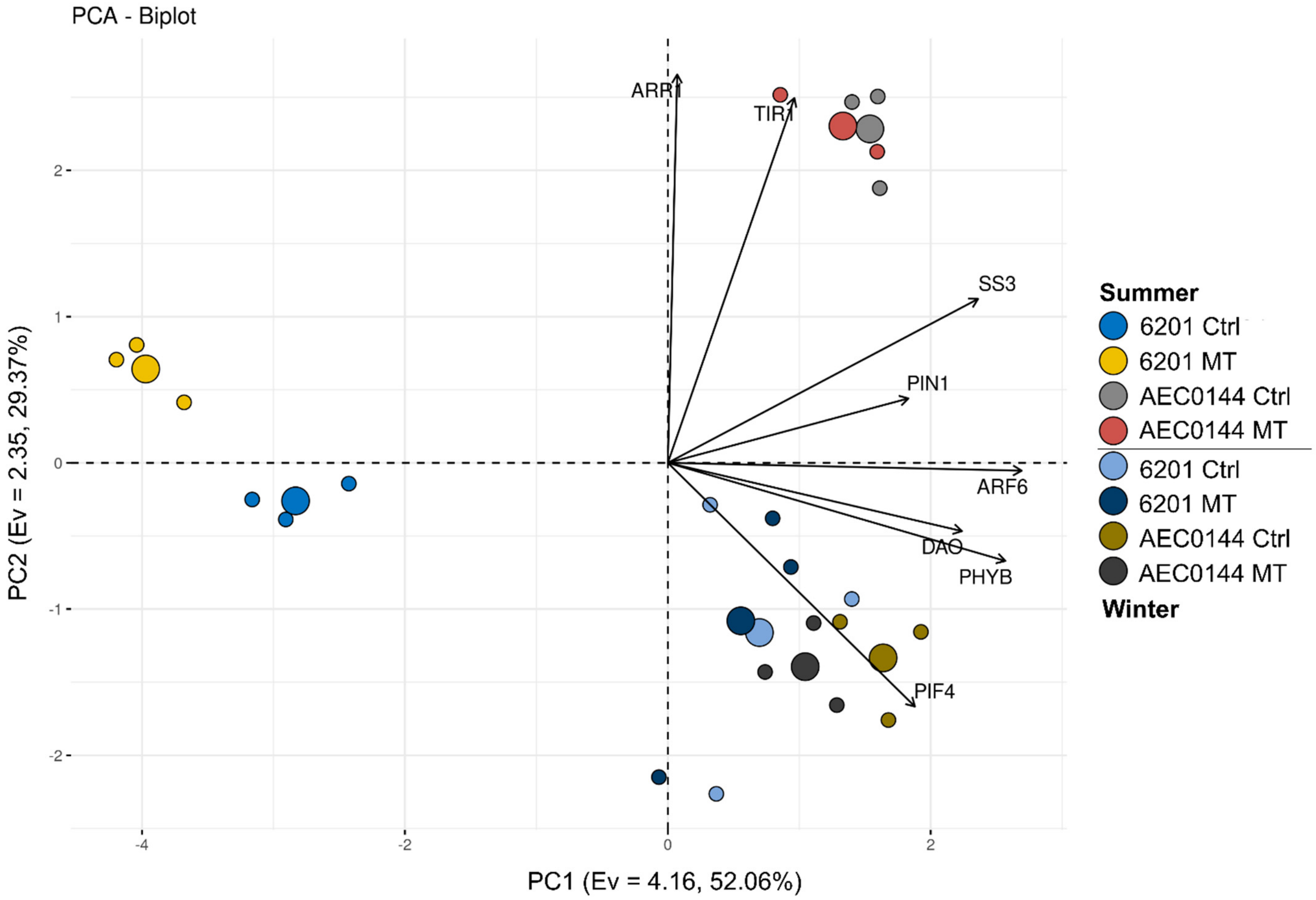

Figure S3. Principal Component Analysis of relative gene expression levels at 7 dpe. Axes represent principal components (PC) and corresponding eigenvalues (Ev) and percentage of variance explained. Regular circles represent biological replicates (n = 3), while larger ones are the centroids for each group indicated in the legend. *Eucalyptus dunnii* 6201 (hardR); *E.* ×*urograndis* AEC0144 (easyR).

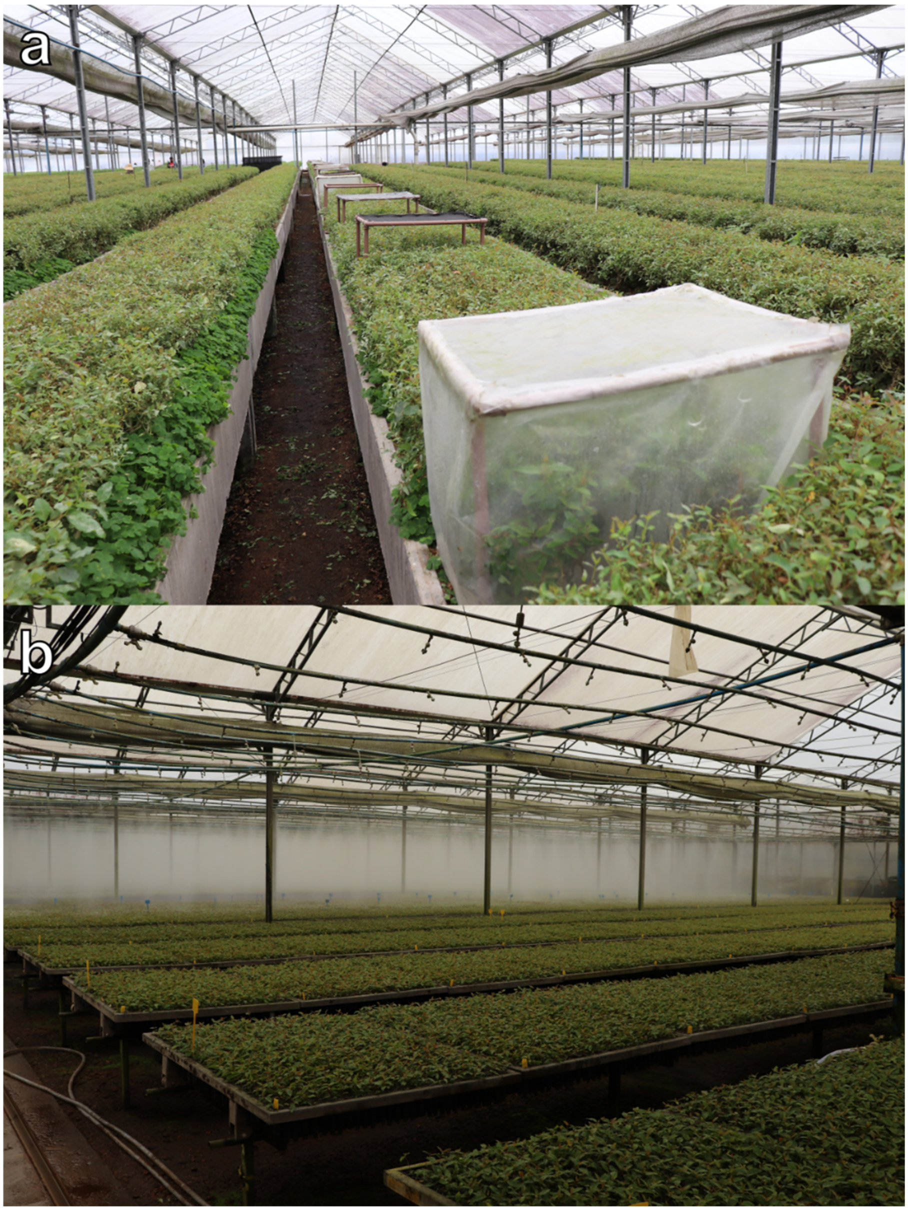

Figure S4. Growth environments used for *Eucalyptus* propagation. (a) Mini-stump growth facility (clonal garden). (b) Rooting greenhouse (fogging irrigation in the background).

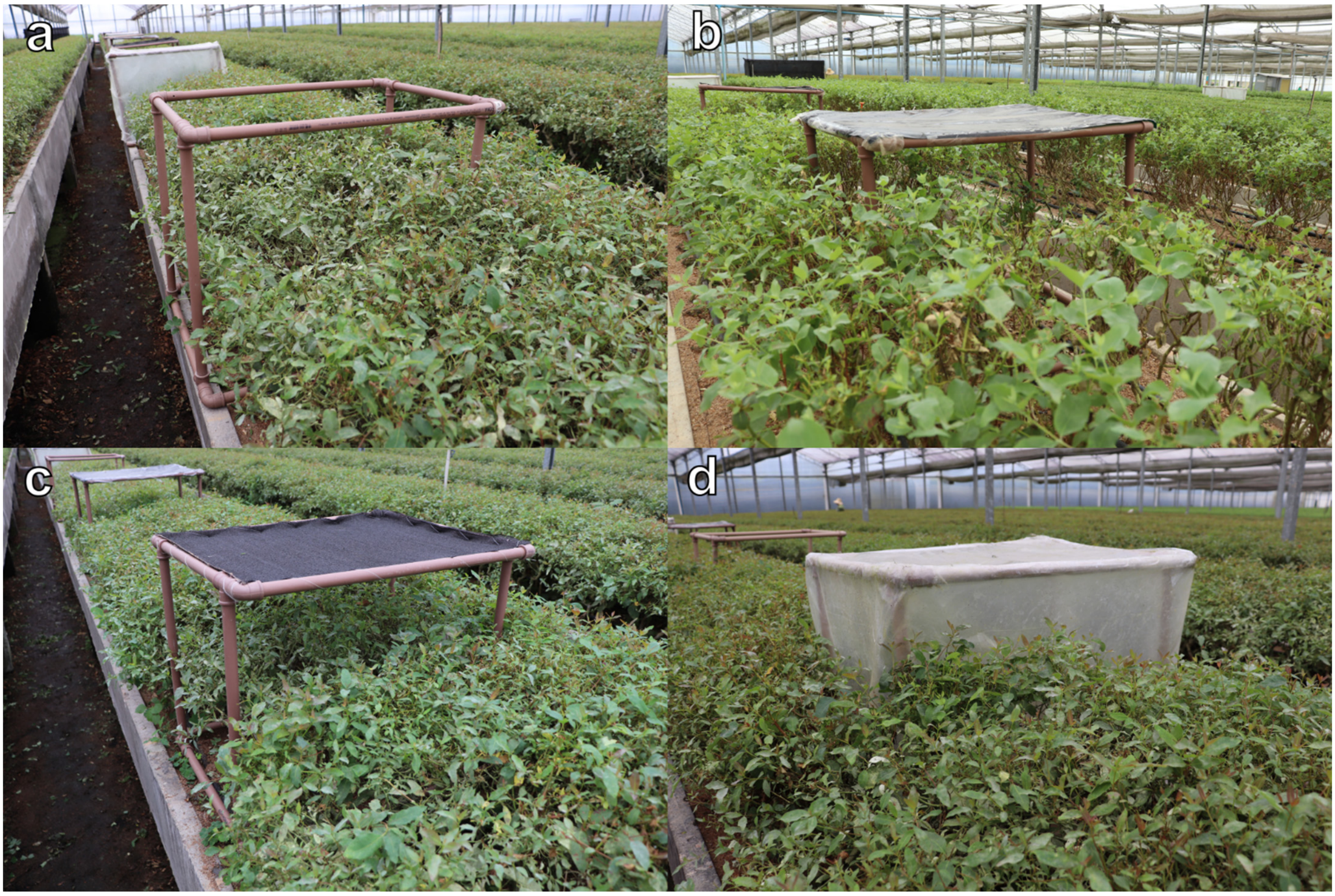

Figure S5. Mini-stump environmental modulation. (a) Control condition (Ctrl). (b) Far-red-enriched light treatment (FR). (c) Shade nets light treatment (SH). (d) Mini-tunnel treatment (MT).

COLOR FILTER TECHNICAL DATA SHEET

SWATCHBOOK: CINELUX
COLOR FILTER: #91 PRIMARY GREEN
DESCRIPTION: Color Effects Lighting Filter.
TRANSMISSION = 7% or -3.8 stop loss
MIRED SHIFT = Not Applicable.
CC EQUIVALENT = Not Applicable.

COLORIMETRIC DATA
OBSERVER: CIE 1964 10° R: 0
SOURCE: • 'A' (tungsten) G: 46
° 'D65' (daylight) B: 35

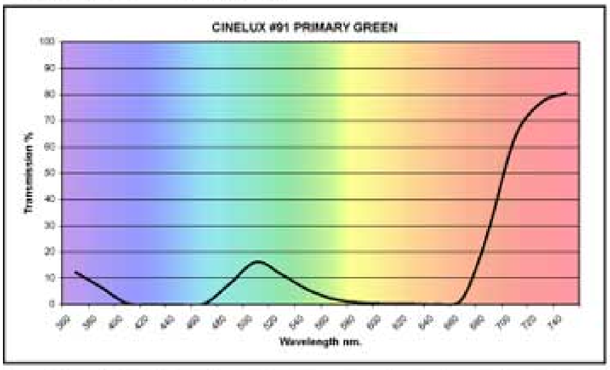

HUNTER LAB SOURCE A
L* 22.888
A* -38.718
B* 4.872

HUNTER LAB SOURCE D65
L* 26.773
A* -56.764
B* 19.1

CIE 1964 SOURCE A
Y 3.768
(x) 0.283
(y) 0.560

CIE 1964 SOURCE D65
Y 5.013
(x) 0.180
(y) 0.571

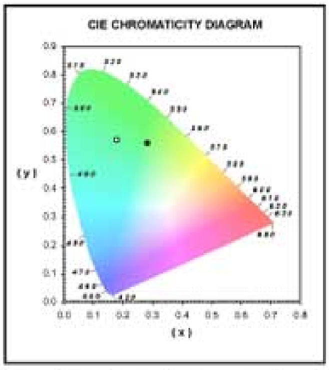

nm.	360	380	400	420	440	460	480	500	520	540	560	580	600	620	640	660	680	700	720	740
trans %	13	7	1	0	0	0	8	16	11	6	2	1	0	0	0	2	26	63	77	81

MATERIAL SPECIFICATIONS:
General Description: Deep-Dyed Polyester Film
Substrate: PET (Polyethylene Terephthalate)
Thickness: 1.5 mil (.0015" or 38 micron)

Manufactured in: U.S.A.

AVAILABLE SIZES:
√ 20 in. x 24 in. sheets (50cm x 60cm)
21 in. x 24 in. sheets (53 x 60cm)
√ 24 in. x 25 ft. rolls (60cm x 7.62m)
√ 48 in. x 25 ft. rolls (121cm x 7.62m)
60 in. x 20 ft. rolls (152.4cm x 6.10m)
13.5 in. Diameter Glass (34.3cm) - Cut to order

Copyright 2001, Rosco Laboratories Inc.
All Rights Reserved.

Figure S6. Primary Green light filter technical data sheet. All rights reserved to Rosco Laboratories Inc.

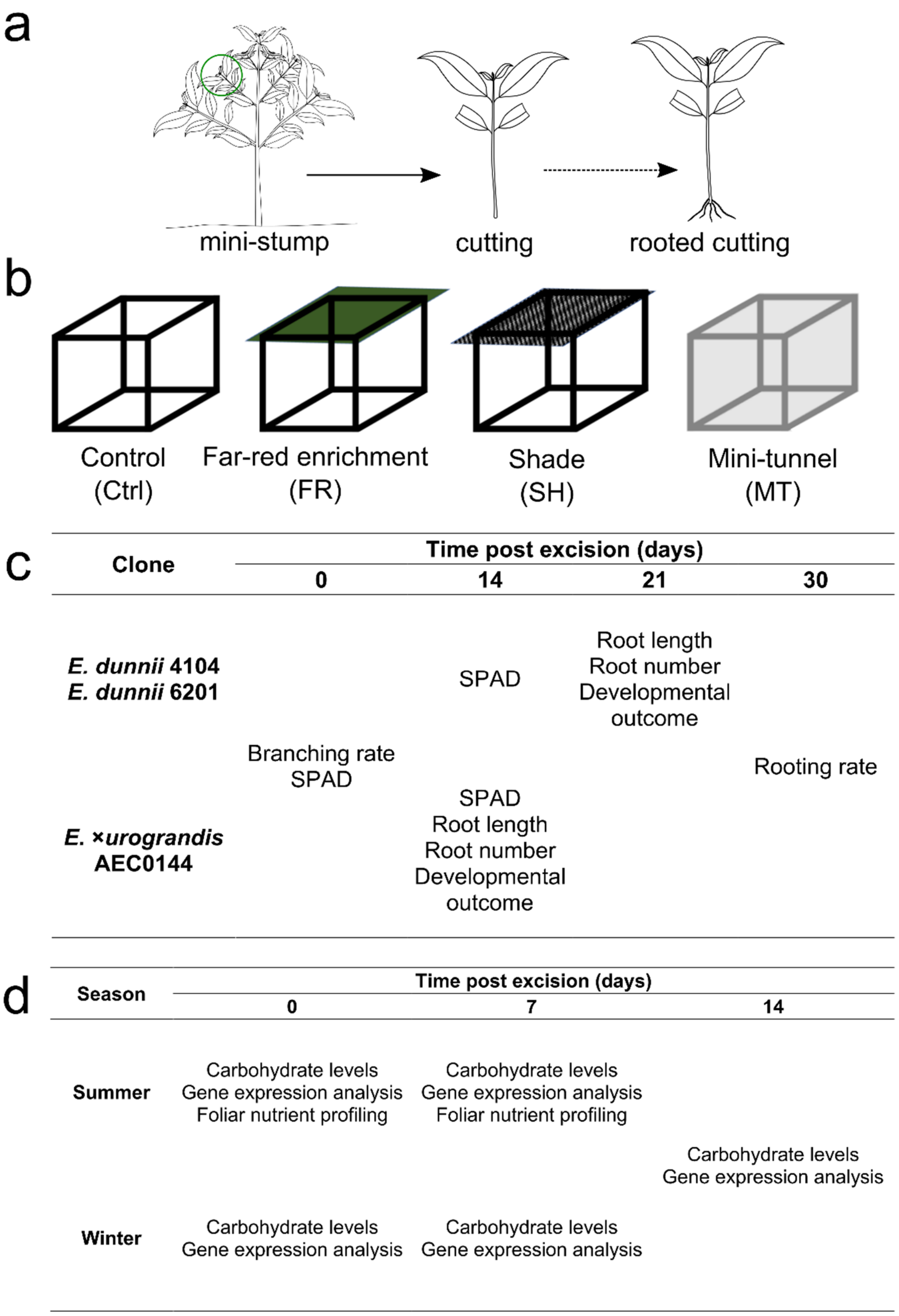

Figure S7. Overview of the propagation experimental design and analyses performed for whole-cutting samples. (a) *Eucalyptus dunnii* 4104 and 6201 (hardR clones) and *E. ×urograndis* AEC0144 (easyR) mini-stumps were cultivated in sand beds and weekly pruned to obtain cuttings, which were transferred to rooting substrate to form adventitious roots. (b) Mini-stumps were subjected to one of four conditions: control (Ctrl), far-red enrichment (FR), shade (SH), and mini-tunnel (MT). (c) For a year, branching and rooting rates were assessed, as well as chlorophyll levels (SPAD) and the developmental outcome of cuttings. Root growth measurements were made at different intervals for different species, due to their different growth rates. (d) *E. dunnii* 6201 and *E. ×urograndis* AEC0144 cuttings were monitored at 0-, 7-, and 14-days post excision, during summer

and winter, through carbohydrate quantification, gene expression analysis, and foliar nutrient profiling.

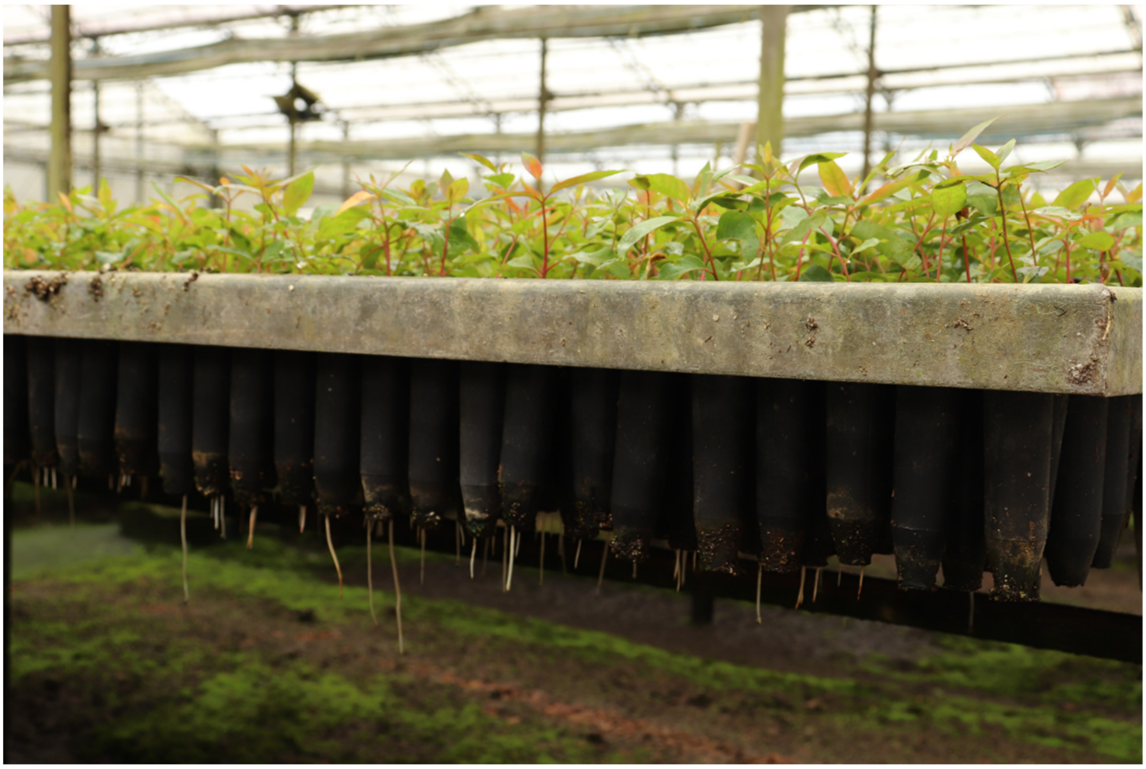

Figure S8. Rooted *Eucalyptus* cuttings on a rooting greenhouse table.

Table S1. Meteorological data for the study site. Values are shown as mean±sd.

Season	Air temperature (°C)*	Relative humidity (%)*	Day length (h)**
Summer	25.1±2.7	77.7±7.7	13.3±0.6
Fall	20.1±2.6	85.1±8.0	10.9±0.6
Winter	15.4±4.1	77.6±8.7	10.9±0.6
Spring	22.2±3.5	71.1±11.5	13.3±0.6

* Hourly measurements. **Daily measurements.

Table S2. List of primers used for gene expression analysis. F = forward primer, R = reverse primer.

**Gene id.**	**Oligonucleotide sequence**
ARF6<br>*AUXIN RESPONSE FACTOR 6*	F: AGTGAACTCGCTCGCATGT<br>R: CGTCACCCAGGAGAAGGATA
ARR1<br>*ARABIDOPSIS TYBE-B RESPONSE REGULATOR*	F: TTCCATGGTCAGCTGCAGTA<br>R: CCAGCACCACCACTTCAAAT
DAO<br>*DIOXYGENASE FOR AUXIN OXIDATION*	F: AGTGATGGACAAGTCGGGTT<br>R: GCTGCCTTCTTTGGACTCAAC
H2B<br>*HISTONE 2B*	F: CGTCTCAGAAGGGACCAAGG<br>R: ACGGGTAACACACAACTTCC
PHYB<br>*PHYTOCHROME B*	F: TGCGTCCAAGTCTGAAGCAA<br>R: TCACCAGGACACACGATCCTAA
PIF4<br>*PHYTOCHROME INTERACTING FACTOR 4*	F: CTCTGAAAGGAGGAGGAGGGAT<br>R: CCAGGAAACATCATCGGAGCC
PIN1<br>*PINFORMED 1*	F: TTTGCCCATAACGCTCGTCT<br>R: CACCATCCCACCATGTCCAA
SAND<br>*SAND-DOMAIN PROTEIN*	F: CCATTCAACACTCTCCGACA<br>R: TGTGTGACCCAGCAGAGTAAT
SS3<br>*STARCH SYNTHASE 3*	F: ATCGTGCTCGCCTTTGTTTG<br>R: CCAAAATGAAATCCGCCCCG
TIR1<br>*TRANSPORT INHIBITION RESPONSE 1*	F: GTGGCACTATGGTGTGGTGA<br>R: AGCTCAGCCAAGTGCAAAAT

Table S3. Pearson's correlation coefficient between gene expression levels at 0 dpe and principal components. Ev: eigenvalues, PC: principal component.

PC (Ev, % variance explained)	*ARF6*	*ARR1*	*DAO*	*PHYB*	*PIF4*	*PIN1*	*SS3*	*TIR1*
PC1 (3.76, 46.95%)		0.55	0.93		0.87	0.58	0.82	
PC2 (2.29, 27.85%)	-0.65	0.71		-0.57				0.86

Table S4. Pearson's correlation coefficient between gene expression levels at 7 dpe and principal components. Ev: eigenvalues, PC: principal component.

PC (Ev, % variance explained)	*ARF6*	*ARR1*	*DAO*	*PHYB*	*PIF4*	*PIN1*	*SS3*	*TIR1*
PC1 (4.16, 52.06%)	0.97		0.80	0.92	0.68	0.66	0.85	
PC2 (2.35, 29.37%)		0.95			-0.60			0.90

Table S5. Pearson's correlation coefficient between gene expression levels at 14 dpe and principal components. Ev: eigenvalues, PC: principal component.

**PC (Ev, % variance explained)**	***ARF6***	***ARR1***	***DAO***	***PHYB***	***PIF4***	***PIN1***	***SS3***	***TIR1***
PC1 (3.36, 41.94%)	0.65		0.82	0.77	0.77	0.83		
PC2 (1.77, 22.11%)		0.72					0.76	-0.66
PC3 (1.03, 12.88%)	-0.62							0.47

Table S6. Pearson's correlation coefficient between foliar nutrient levels and principal components. Ev: eigenvalues, PC: principal component.

PC (Ev, % variance explained)	N	P	K	Ca	Mg	S	B	Cu	Fe	Mn	Zn	Na	Al
PC1 (5.49, 42.23%)	0.90	0.92	0.76	0.84	0.77	-0.55			-0.61	0.66			-0.67
PC2 (2.93, 22.56%)				0.42			0.84	0.91			0.94		
PC3 (1.35, 10.41%)						0.60						0.80	

Table S7. Productivity data ANOVA table. DFn: degrees of freedom in the numerator, DFd: degrees of freedom in the denominator, ges: generalized eta squared, sp: species, ttm: treatment.

**Branching rate**						
**Effect**	**DFn**	**DFd**	**F**	**p**	**p<.05**	**ges**
season	3	713	126.807	7.93e-66	*	0.348
sp	2	713	25.237	2.57e-11	*	0.066
ttm	3	713	24.342	5.27e-15	*	0.093
season:sp	6	713	7.379	1.13e-07	*	0.058
season:ttm	9	713	1.114	3.50e-01		0.014
sp:ttm	6	713	3.385	3.00e-03	*	0.028
season:sp:ttm	18	713	1.129	3.18e-01		0.028
**Rooting rate**						
**Effect**	**DFn**	**DFd**	**F**	**p**	**p<.05**	**ges**
season	3	2636	184.837	8.94e-109	*	0.174
sp	2	2636	2255.684	0.00e+00	*	0.631
ttm	3	2636	4.496	4.00e-03	*	0.005
season:sp	6	2636	53.474	1.86e-62	*	0.109
season:ttm	9	2636	8.638	7.22e-13	*	0.029
sp:ttm	6	2636	4.691	9.33e-05	*	0.011
season:sp:ttm	18	2636	17.528	8.13e-53	*	0.107
**Overall productivity**						
**Effect**	**DFn**	**DFd**	**F**	**p**	**p<.05**	**ges**
season	3	705	297.049	1.27e-124	*	0.558
sp	2	705	501.045	4.13e-136	*	0.587
ttm	3	705	21.145	3.99e-13	*	0.083
season:sp	6	705	39.361	2.36e-41	*	0.251
season:ttm	9	705	4.636	5.58e-06	*	0.056
sp:ttm	6	705	4.703	1.05e-04	*	0.038
season:sp:ttm	18	705	1.463	9.60e-02		0.036

Table S8. Carbohydrate content ANOVA table. DFn: degrees of freedom in the numerator, DFd: degrees of freedom in the denominator, ges: generalized eta squared, sp: species, ttm: treatment, dpe: time post excision.

Soluble sugar content						
**Effect**	**DFn**	**DFd**	**F**	**p**	**p<.05**	**ges**
season	1	48	3.112	8.40e-02		0.061000
sp	1	48	18.414	8.57e-02	*	0.277000
ttm	1	48	3.208	8.00e-02		0.063000
dpe	2	48	11.487	8.38e-05	*	0.324000
season:sp	1	48	39.183	1.00e-07	*	0.449000
season:ttm	1	48	1.411	2.41e-01		0.029000
sp:ttm	1	48	0.033	8.56e-01		0.000696
season:dpe	2	48	2.458	9.60e-02		0.093000
sp:dpe	2	48	1.804	1.76e-01		0.070000
ttm:dpe	2	48	1.154	3.24e-01		0.046000
season:sp:ttm	1	48	2.179	1.46e-01		0.043000
season:sp:dpe	2	48	4.290	1.90e-02	*	0.152000
season:ttm:dpe	2	48	0.618	5.43e-01		0.025000
sp:ttm:dpe	2	48	1.381	2.61e-01		0.054000
season:sp:ttm:dpe	2	48	2.332	1.08e-01		0.089000
**Starch content**						
**Effect**	**DFn**	**DFd**	**F**	**p**	**p<.05**	**ges**
season	1	48	14.582	0.000385	*	2.33e-01
sp	1	48	0.002	0.969000		3.23e-05
ttm	1	48	0.044	0.835000		9.14e-04
dpe	2	48	3.169	0.051000		1.17e-01
season:sp	1	48	17.949	0.000102	*	2.72e-01
season:ttm	1	48	1.255	0.268000		2.50e-01
sp:ttm	1	48	2.637	0.111000		5.20e-02
season:dpe	2	48	3.155	0.052000		1.16e-01
sp:dpe	2	48	4.832	0.012000	*	1.68e-01
ttm:dpe	2	48	0.708	0.498000		2.90e-02
season:sp:ttm	1	48	2.583	0.115000		5.10e-02
season:sp:dpe	2	48	1.752	0.184000		6.80e-02
season:ttm:dpe	2	48	0.247	0.782000		1.00e-02
sp:ttm:dpe	2	48	0.714	0.495000		2.90e-02
season:sp:ttm:dpe	2	48	0.750	0.478000		3.00e-02

Table S9. RT-qPCR data ANOVA table. DFn: degrees of freedom in the numerator, DFd: degrees of freedom in the denominator, ges: generalized eta squared, sp: species, ttm: treatment, dpe: time post excision.

ARR1 expression levels						
**Effect**	**DFn**	**DFd**	**F**	**p**	**p<.05**	**ges**
season	1	48	31.478	9.81e-07	*	3.96e-01
sp	1	48	7.687	8.00e-03	*	1.38e-01
ttm	1	48	4.614	3.70e-02	*	8.80e-02
dpe	2	48	0.711	4.96e-01		2.90e-02
season:sp	1	48	56.881	1.09e-09	*	5.42e-01
season:ttm	1	48	0.010	9.20e-01		2.12e-04
sp:ttm	1	48	0.149	7.02e-01		3.00e-03
season:dpe	2	48	8.341	7.78e-04	*	2.58e-01
sp:dpe	2	48	4.111	2.20e-02	*	1.46e-01
ttm:dpe	2	48	1.475	2.39e-01		5.80e-02
season:sp:ttm	1	48	0.003	9.60e-01		5.35e-05
season:sp:dpe	2	48	2.512	9.20e-02		9.50e-02
season:ttm:dpe	2	48	1.462	2.42e-01		5.70e-02
sp:ttm:dpe	2	48	0.185	8.32e-01		8.00e-03
season:sp:ttm:dpe	2	48	0.717	4.93e-01		2.90e-02
DAO expression levels						
**Effect**	**DFn**	**DFd**	**F**	**p**	**p<.05**	**ges**
season	1	48	45.983	1.59e-08	*	4.89e-01
sp	1	48	15.161	3.05e-04	*	2.40e-01
ttm	1	48	2.625	1.12e-01		5.20e-02
dpe	2	48	11.571	7.92e-05	*	3.25e-01
season:sp	1	48	17.033	1.45e-04	*	2.62e-01
season:ttm	1	48	0.104	7.49e-01		2.00e-03
sp:ttm	1	48	0.004	9.50e-01		8.25e-05
season:dpe	2	48	9.559	3.20e-04	*	2.85e-01
sp:dpe	2	48	1.330	2.74e-01		5.30e-02
ttm:dpe	2	48	0.926	4.03e-01		3.70e-02
season:sp:ttm	1	48	0.002	9.63e-01		4.56e-05
season:sp:dpe	2	48	8.486	6.99e-04	*	2.61e-01
season:ttm:dpe	2	48	5.043	1.00e-02	*	1.74e-01
sp:ttm:dpe	2	48	4.502	1.60e-02	*	1.58e-01
season:sp:ttm:dpe	2	48	0.027	9.73e-01		1.00e-03
PHYB expression levels						
**Effect**	**DFn**	**DFd**	**F**	**p**	**p<.05**	**ges**
season	1	48	99.392	2.80e-13	*	0.674000
sp	1	48	27.384	3.63e-06	*	0.363000
ttm	1	48	0.033	8.56e-01		0.000695
dpe	2	48	6.257	4.00e-03	*	0.207000
season:sp	1	48	5.260	2.60e-02	*	0.099000
season:ttm	1	48	0.302	5.85e-01		0.006000
sp:ttm	1	48	0.021	8.85e-01		0.000439
season:dpe	2	48	1.330	2.74e-01		0.053000
sp:dpe	2	48	3.445	4.00e-02	*	0.126000
ttm:dpe	2	48	1.333	2.73e-01		0.053000

season:sp:ttm	1	48	0.015	9.04e-01		0.000307
season:sp:dpe	2	48	13.436	2.32e-05	*	0.359000
season:ttm:dpe	2	48	2.630	8.20e-02		0.099000
sp:ttm:dpe	2	48	0.540	5.68e-01		0.022000
season:sp:ttm:dpe	2	48	0.467	6.30e-01		0.019000
***PIF4* expression levels**						
**Effect**	**DFn**	**DFd**	**F**	**p**	**p<.05**	**ges**
season	1	48	129.082	3.30e-15	*	0.72900
sp	1	48	41.486	5.30e-08	*	0.46400
ttm	1	48	0.629	4.32e-01		0.01300
dpe	2	48	2.071	1.37e-01		0.07900
season:sp	1	48	7.914	7.00e-03	*	0.14200
season:ttm	1	48	0.491	4.87e-01		0.01000
sp:ttm	1	48	0.475	4.94e-01		0.01000
season:dpe	2	48	0.109	8.97e-01		0.00500
sp:dpe	2	48	4.247	2.00e-02	*	0.15000
ttm:dpe	2	48	4.061	2.30e-02	*	0.14500
season:sp:ttm	1	48	0.030	8.63e-01		0.00063
season:sp:dpe	2	48	34.709	4.74e-10	*	0.59100
season:ttm:dpe	2	48	0.247	7.82e-01		0.01000
sp:ttm:dpe	2	48	0.906	4.11e-01		0.03600
season:sp:ttm:dpe	2	48	4.458	1.70e-02	*	0.15700
***PIN1* expression levels**						
**Effect**	**DFn**	**DFd**	**F**	**p**	**p<.05**	**ges**
season	1	48	34.322000	4.12e-07	*	4.17e-01
sp	1	48	6.712000	1.30e-02	*	1.23e-01
ttm	1	48	4.740000	3.40e-02	*	9.00e-02
dpe	2	48	0.726000	4.89e-01		2.90e-02
season:sp	1	48	59.220000	6.37e-10	*	5.52e-01
season:ttm	1	48	0.024000	8.76e-01		5.10e-04
sp:ttm	1	48	0.223000	6.39e-01		5.00e-03
season:dpe	2	48	9.630000	3.04e-04	*	2.86e-01
sp:dpe	2	48	4.173000	2.10e-02	*	1.48e-01
ttm:dpe	2	48	1.801000	1.76e-01		7.00e-02
season:sp:ttm	1	48	0.000316	9.86e-01		6.59e-06
season:sp:dpe	2	48	3.239000	4.80e-02	*	1.19e-01
season:ttm:dpe	2	48	1.731000	1.88e-01		6.70e-02
sp:ttm:dpe	2	48	0.177000	8.38e-01		7.00e-03
season:sp:ttm:dpe	2	48	0.677000	5.13e-01		2.70e-02
***SS3* expression levels**						
**Effect**	**DFn**	**DFd**	**F**	**p**	**p<.05**	**ges**
season	1	48	0.120	7.31e-01		0.002
sp	1	48	93.168	7.96e-13	*	0.660
ttm	1	48	0.099	7.55e-01		0.002
dpe	2	48	5.167	9.00e-03	*	0.177
season:sp	1	48	8.191	6.00e-03	*	0.146
season:ttm	1	48	1.158	2.87e-01		0.024
sp:ttm	1	48	0.125	7.25e-01		0.003
season:dpe	2	48	1.489	2.36e-01		0.058

sp:dpe	2	48	3.834	2.90e-02	*	0.138
ttm:dpe	2	48	0.541	5.86e-01		0.022
season:sp:ttm	1	48	0.506	4.80e-01		0.010
season:sp:dpe	2	48	18.183	1.32e-06	*	0.431
season:ttm:dpe	2	48	0.855	4.31e-01		0.034
sp:ttm:dpe	2	48	1.070	3.51e-01		0.043
season:sp:ttm:dpe	2	48	2.110	1.32e-01		0.081
***TIR1* expression levels**						
**Effect**	**DFn**	**DFd**	**F**	**p**	**p<.05**	**ges**
season	1	48	551.135	5.38e-28	*	0.920
sp	1	48	17.680	1.13e-04	*	0.269
ttm	1	48	1.376	2.47e-01		0.028
dpe	2	48	4.982	1.10e-02	*	0.172
season:sp	1	48	3.324	7.50e-02		0.065
season:ttm	1	48	0.380	5.40e-01		0.008
sp:ttm	1	48	0.393	5.34e-01		0.008
season:dpe	2	48	1.573	2.18e-01		0.062
sp:dpe	2	48	9.363	3.69e-04	*	0.281
ttm:dpe	2	48	1.116	3.36e-01		0.044
season:sp:ttm	1	48	0.275	6.02e-01		0.006
season:sp:dpe	2	48	6.825	2.00e-03	*	0.221
season:ttm:dpe	2	48	1.100	3.41e-01		0.044
sp:ttm:dpe	2	48	1.572	2.18e-01		0.061
season:sp:ttm:dpe	2	48	1.701	1.93e-01		0.066

Table S10. Nutrient profile ANOVA table. DFn: degrees of freedom in the numerator, DFd: degrees of freedom in the denominator, ges: generalized eta squared, sp: species, ttm: treatment, dpe: time post excision.

			**N content**			
**Effect**	**DFn**	**DFd**	**F**	**p**	**p<.05**	**ges**
sp	1	16	1.996	1.77e-01		0.111
ttm	1	16	0.426	5.23e-01		0.026
dpe	1	16	77.713	1.54e-07	*	0.829
sp:ttm	1	16	0.975	3.38e-01		0.057
sp:dpe	1	16	9.229	8.00e-03	*	0.366
ttm:dpe	1	16	0.165	6.90e-01		0.010
sp:ttm:dpe	1	16	0.032	8.61e-01		0.002
			**P content**			
**Effect**	**DFn**	**DFd**	**F**	**p**	**p<.05**	**ges**
sp	1	19	0.835	3.72e-01		0.042000
ttm	1	19	0.009	9.24e-01		0.000489
dpe	1	19	55.255	4.90e-07	*	0.744000
sp:ttm	1	19	0.131	7.21e-01		0.007000
sp:dpe	1	19	4.077	5.80e-02		0.177000
ttm:dpe	1	19	0.899	3.55e-01		0.045000
sp:ttm:dpe	1	19	0.562	4.62e-01		0.029000
			**K content**			
**Effect**	**DFn**	**DFd**	**F**	**p**	**p<.05**	**ges**
sp	1	17	1.184	0.292000		0.065
ttm	1	17	1.012	0.329000		0.056
dpe	1	17	23.195	0.000161	*	0.577
sp:ttm	1	17	3.127	0.095000		0.155
sp:dpe	1	17	0.310	0.585000		0.018
ttm:dpe	1	17	2.059	0.169000		0.108
sp:ttm:dpe	1	17	12.697	0.002000	*	0.428
			**Ca content**			
**Effect**	**DFn**	**DFd**	**F**	**p**	**p<.05**	**ges**
sp	1	17	1.890	1.87e-01		0.100
ttm	1	17	1.380	2.56e-01		0.075
dpe	1	17	67.611	2.51e-07	*	0.799
sp:ttm	1	17	5.916	2.60e-02	*	0.258
sp:dpe	1	17	0.437	5.17e-01		0.025
ttm:dpe	1	17	28.100	5.87e-05	*	0.623
sp:ttm:dpe	1	17	1.029	3.25e-01		0.057
			**Mg content**			
**Effect**	**DFn**	**DFd**	**F**	**p**	**p<.05**	**ges**
sp	1	17	0.085	7.74e-01		0.005000
ttm	1	17	0.015	9.03e-01		0.000908
dpe	1	17	18.059	5.41e-04	*	0.515000
sp:ttm	1	17	0.181	6.76e-01		0.011000
sp:dpe	1	17	0.011	9.17e-01		0.000651
ttm:dpe	1	17	25.862	9.17e-05	*	0.603000
sp:ttm:dpe	1	17	0.158	6.96e-01		0.009000

**S content**						
**Effect**	**DFn**	**DFd**	**F**	**p**	**p<.05**	**ges**
sp	1	16	0.965	0.341		0.057
ttm	1	16	2.018	0.175		0.112
dpe	1	16	8.955	0.009	*	0.359
sp:ttm	1	16	7.401	0.015	*	0.316
sp:dpe	1	16	0.716	0.410		0.043
ttm:dpe	1	16	0.167	0.689		0.010
sp:ttm:dpe	1	16	1.285	0.274		0.074
**B content**						
**Effect**	**DFn**	**DFd**	**F**	**p**	**p<.05**	**ges**
sp	1	16	8.588	0.010	*	0.349
ttm	1	16	6.705	0.020	*	0.295
dpe	1	16	8.632	0.010	*	0.350
sp:ttm	1	16	8.451	0.010	*	0.346
sp:dpe	1	16	9.711	0.007	*	0.379
ttm:dpe	1	16	10.341	0.005	*	0.393
sp:ttm:dpe	1	16	14.386	0.002	*	0.473
**Cu content**						
**Effect**	**DFn**	**DFd**	**F**	**p**	**p<.05**	**ges**
sp	1	16	0.979	0.337		0.058
ttm	1	16	1.327	0.266		0.077
dpe	1	16	8.160	0.011	*	0.338
sp:ttm	1	16	6.868	0.019	*	0.300
sp:dpe	1	16	1.072	0.316		0.063
ttm:dpe	1	16	1.691	0.212		0.096
sp:ttm:dpe	1	16	6.482	0.022	*	0.288
**Fe content**						
**Effect**	**DFn**	**DFd**	**F**	**p**	**p<.05**	**ges**
sp	1	17	0.037	0.850		0.002
ttm	1	17	0.523	0.479		0.030
dpe	1	17	6.103	0.024	*	0.264
sp:ttm	1	17	1.625	0.220		0.087
sp:dpe	1	17	6.054	0.025	*	0.263
ttm:dpe	1	17	0.436	0.518		0.025
sp:ttm:dpe	1	17	4.539	0.048	*	0.211
**Mn content**						
**Effect**	**DFn**	**DFd**	**F**	**p**	**p<.05**	**ges**
sp	1	16	20.449	3.47e-04	*	0.561
ttm	1	16	19.502	4.33e-04	*	0.549
dpe	1	16	38.455	1.27e-05	*	0.706
sp:ttm	1	16	0.904	3.56e-01		0.053
sp:dpe	1	16	0.046	8.33e-01		0.003
ttm:dpe	1	16	0.850	3.70e-01		0.050
sp:ttm:dpe	1	16	1.702	2.10e-01		0.096
**Zn content**						
**Effect**	**DFn**	**DFd**	**F**	**p**	**p<.05**	**ges**
sp	1	18	0.627	0.439		0.034
ttm	1	18	0.961	0.340		0.051

dpe	1	18	7.811	0.012	*	0.303
sp:ttm	1	18	5.794	0.027	*	0.243
sp:dpe	1	18	1.188	0.290		0.062
ttm:dpe	1	18	1.375	0.256		0.071
sp:ttm:dpe	1	18	5.926	0.026	*	0.248
			**Na content**			
**Effect**	**DFn**	**DFd**	**F**	**p**	**p<.05**	**ges**
sp	1	17	5.012	0.039	*	0.228
ttm	1	17	0.852	0.369		0.048
dpe	1	17	0.421	0.525		0.024
sp:ttm	1	17	1.928	0.183		0.102
sp:dpe	1	17	0.133	0.720		0.008
ttm:dpe	1	17	0.242	0.629		0.014
sp:ttm:dpe	1	17	3.278	0.088		0.162
			**Al content**			
**Effect**	**DFn**	**DFd**	**F**	**p**	**p<.05**	**ges**
sp	1	16	0.071000	0.793000		4.00e-03
ttm	1	16	3.134000	0.096000		1.64e-01
dpe	1	16	17.535000	0.000696	*	5.23e-01
sp:ttm	1	16	0.084000	0.775000		5.00e-03
sp:dpe	1	16	0.525000	0.479000		3.20e-02
ttm:dpe	1	16	1.050000	0.321000		6.20e-02
sp:ttm:dpe	1	16	0.000503	0.982000		3.15e-05
